# PhenoDEF: a corpus for annotating sentences with information of phenotype definitions in biomedical literature

**DOI:** 10.1186/s13326-022-00272-6

**Published:** 2022-06-11

**Authors:** Samar Binkheder, Heng-Yi Wu, Sara K. Quinney, Shijun Zhang, Md. Muntasir Zitu, Chien‐Wei Chiang, Lei Wang, Josette Jones, Lang Li

**Affiliations:** 1 Department of Biohealth Informatics, Indiana University School of Informatics and Computing, Indianapolis, IN USA; 2 Medical Informatics Unit, Department of Medical Education, College of Medicine, King Saud University, Riyadh, Saudi Arabia; 3 Development Science Informatics, Genentech, South San Francisco, CA USA; 4 Department of Obstetrics and Gynecology, Indiana University School of Medicine, Indianapolis, IN USA; 5 Department of Biomedical Informatics, College of Medicine, The Ohio State University, Columbus, OH USA; 6 250 Lincoln Tower, 1800 Cannon Drive, Columbus, OH 43210 USA

**Keywords:** Adverse drug events, Biomedical corpus, Electronic health records, Phenotype definitions, Text mining

## Abstract

**Background:**

Adverse events induced by drug-drug interactions are a major concern in the United States. Current research is moving toward using electronic health record (EHR) data, including for adverse drug events discovery. One of the first steps in EHR-based studies is to define a phenotype for establishing a cohort of patients. However, phenotype definitions are not readily available for all phenotypes. One of the first steps of developing automated text mining tools is building a corpus. Therefore, this study aimed to develop annotation guidelines and a gold standard corpus to facilitate building future automated approaches for mining phenotype definitions contained in the literature. Furthermore, our aim is to improve the understanding of how these published phenotype definitions are presented in the literature and how we annotate them for future text mining tasks.

**Results:**

Two annotators manually annotated the corpus on a sentence-level for the presence of evidence for phenotype definitions. Three major categories (inclusion, intermediate, and exclusion) with a total of ten dimensions were proposed characterizing major contextual patterns and cues for presenting phenotype definitions in published literature. The developed annotation guidelines were used to annotate the corpus that contained 3971 sentences: 1923 out of 3971 (48.4%) for the inclusion category, 1851 out of 3971 (46.6%) for the intermediate category, and 2273 out of 3971 (57.2%) for exclusion category. The highest number of annotated sentences was 1449 out of 3971 (36.5%) for the “Biomedical & Procedure” dimension. The lowest number of annotated sentences was 49 out of 3971 (1.2%) for “The use of NLP”. The overall percent inter-annotator agreement was 97.8%. Percent and Kappa statistics also showed high inter-annotator agreement across all dimensions.

**Conclusions:**

The corpus and annotation guidelines can serve as a foundational informatics approach for annotating and mining phenotype definitions in literature, and can be used later for text mining applications.

**Supplementary Information:**

The online version contains supplementary material available at 10.1186/s13326-022-00272-6.

## Background

Adverse events induced by drug-drug interactions are a major concern in the United States [1]. The U.S. Food and Drug Administration (FDA) reported around 297,010 serious outcomes and around 44,693 deaths due to adverse drug events (ADEs) in the first quarter of 2017 [2]. The current direction is moving towards the utilization of electronic health records (EHRs) for clinical research, including ADE discovery [3–6]. EHR-based research, in general, relies on the process of electronic phenotyping to advance knowledge of a disease or an adverse event [7, 8]. An accurate phenotype definition is critical to identifying patients with a certain phenotype from the EHRs [7–10]. A “phenotype” can refer to observable patient characteristics inferred from clinical data [7, 11–13] or drug-related adverse events or reactions [14]. Several methods can be used for EHR electronic phenotyping by utilizing either structured or unstructured data [11, 15, 16], including natural language processing (NLP), rule-based systems, statistical analysis, data mining, machine learning, and hybrid systems [13, 15]. However, it can be challenging to develop new phenotype definitions for each phenotype of interest. These phenotype definitions are present in literature; however, to our knowledge, no work has previously annotated phenotype definitions from full-text publications on a sentence-level for the goal of text mining applications.

### Phenotype definitions

Different institutions view a phenotype definition or a phenotyping case definition differently. For example, Strategic Health IT Advanced Research Projects (SHARP) [17], which is a collaboration effort (academic and industries partners) to advance the secondary use of clinical data, views a phenotype definition as the “inclusion and exclusion criteria for clinical trials, the numerator and denominator criteria for clinical quality metrics, epidemiologic criteria for outcomes research or observational studies, and trigger criteria for clinical decision support rules, among others” [17]. On the other hand, the Electronic Medical Records & Genomics (eMERGE) phenotype definitions extend to include practices as the “algorithmic recognition of any cohort within EHR for a defined purpose. These purposes were inspired by the algorithmic identification of research phenotypes” [17]. Further practices that eMERGE used in developing phenotype definitions include other data modalities, such as diagnosis fields, laboratory values, medication use, and NLP [17]. Here, we include summarized examples of definitions for a phenotype definition, which are:

- ▪ Inclusion and exclusion criteria are performed using the EHR’s structured data and unstructured clinical text to identify a cohort of patients from the EHR [18].
- ▪ EHR-based research is concerned with cohort selection which is the identification of cases and controls for a phenotype of interest. A phenotype definition is developed by combining EHR data, such as billing codes, medications, narrative notes, and laboratory data [19–22].
- ▪ The process of deriving a cohort of a phenotype of interest using either low-throughput or high-throughput approaches [23].
- ▪ The identification of the cohort utilizing risk factors and clinical or medical characteristics and complications [24, 25].

Developing a new phenotype definition can be done either by creating new case definitions or utilizing existing case definitions' information that is already available in existing data sources. Traditional expert-driven phenotyping relies on expert knowledge; however, these definitions might change over time [11]. In addition, this task is challenging due to the complexity of EHRs and the heterogeneity of patient records [15]. Depending on the phenotype of interest as well as the study purpose, standard queries for defining a phenotype can consist of any of the following: logical operators, standardized codes, data fields, and values sets (concepts derived from vocabularies or data standards) [7]. Furthermore, it is also a labor-intensive process in which a multidisciplinary team is needed with experts including biostatisticians, clinical researchers, informaticians, and NLP experts [21]. One example of an expert-driven definition is a study that identified patients with chronic rhinosinusitis (CRS) for a better understanding of the “prevalence, pathophysiology, morbidity, and management” using EHR data [26]. The authors developed a phenotype algorithm to define CRS cases using the International Classification of Diseases, Ninth Revision (ICD-9) diagnosis codes [27] and the Current Procedural Terminology (CPT) codes [28]. The process took several iterations until they achieved a predictive positive value of 91%. Further, they stated that the manual review of sinus computed tomography (CT) results and notes, which was completed by two reviewers in 40 h, was not scalable to larger numbers of patients or notes. Not to mention, their CRS definition has only been tested on one site and its performance is not known in other centers [26]. This creates further difficulties when creating new definitions.

Lessons learned from the eMERGE Network [29] showed that the process of developing, creating, and validating a phenotype definition for a single disease is time-consuming and can take around 6 to 8 months. Consequently, the eMERGE network developed the Phenotype KnowledgeBase (PheKB) [9], which is a phenotype knowledgebase collaborative environment that allows collaborating and commenting between groups of researchers who are invited by a primary author. The PheKB [9] uses an expert-driven approach where new phenotype definitions are generated by multi-institutional inputs and are publicly available for use. The PheKB provides a library of definitions for several phenotypes that include drug response phenotypes such as adverse effects or efficacy, diseases or syndromes, and other traits. Inspired by PheKB modalities or methods [9], a phenotype definition includes the presence of the following attributes: biomedical and procedure information, standard codes, medications, laboratories, and NLP. The NLP has been used in many phenotypes in the PheKB, such as angiotensin-converting enzyme inhibitor (ACE-I) induced cough which provides a list of terms that can be used to identify cases [9]. On the other hand, data and study design can still be important to capture, but these are not the primary modalities/attributes of a phenotype definition.

Another method relies on deriving phenotype definitions from existing data sources, such as EHR and biomedical literature. Some of these have been addressed manually using systematic reviews [30–36] or automatically using computational approaches. Systematic reviews have a big role in medical knowledge; however, with the massive amount of information, there is still a need to use automated approaches to extract medical knowledge. For example, the rate of published clinical trial articles is over 20,000 per year while around 3,000 systematic reviews were indexed in MEDLINE yearly. Overall, conducting systematic reviews can be time-consuming and labor-intensive [37]. On the other hand, the automated approaches for mining phenotypes in the literature are mostly focused on extracting phenotype terminologies [38–40]. This approach can miss important phenotype definitions information that is contained within text sources. Additionally, some of these studies [40, 41] have addressed only one phenotype at a time which might not be generalizable, especially when working on a large-scale set of phenotypes. Furthermore, these studies utilized abstracts rather than full-text articles [40, 41]. Unlike full-text articles that are richer in information, abstracts are not sufficient for the granularity of phenotype definitions information. In addition, such approaches might not be generalizable, especially when working on a large-scale set of phenotypes. In the study done by Botsis and Ball [41], they developed a corpus and a classifier to automate the extraction of “anaphylaxis” definitions from the literature. However, Botsis and Ball [41] only relied on abstracts rather than full-text articles and only addressed one condition, "anaphylaxis". Even though they focused on some features of phenotype definitions, e.g. signs and symptoms, they did not consider other features, such as standardized codes and laboratory measures [41]. Therefore, this effort did not address our information needs that reflect modalities of phenotype definitions such as those used in PheKB.

### Applications of electronic phenotyping and phenotype definitions

Electronic phenotyping is the process of identifying patients with an outcome of interest, such as patients with ADEs [15]. There are two major types of research in the biomedical domain: primary research that directly collects data and secondary research that relies on published information or sources of data. EHR phenotyping is not limited to but is mostly needed in primary research which includes observational studies, also called epidemiological studies. For example, the design of observational studies can include cross-sectional, retrospective, and prospective cohorts [42], where phenotype definitions can be used [15]. Furthermore, other examples of studies that use phenotype definitions are pharmacovigilance, predictive modeling, clinical effectiveness research, and risk factor studies. More examples are shown in Banda et al. research [15]. For a phenotype of interest, different study designs require different cohort designs as well as phenotype definitions where one phenotype can be defined in different ways depending on the study's needs. For instance, type 2 diabetes mellitus can be defined as “simple as patients with type 2 diabetes or far more nuanced, such as patients with stage II prostate cancer and urinary urgency without evidence of urinary tract infection” [15].

New research, such as pharmacovigilance, is moving towards the emergence of electronic health information, machine learning, and NLP [43]. Methods used for electronic phenotyping, include NLP, machine learning, rule-based, and collaborative frameworks [15]. EHRs provide complementary data with some flexibility in extended period tracking, large sample size, and data heterogeneity [24]. The availability of a cohort can create several opportunities for data mining and modeling such as building risk models, detecting ADEs, measuring the effectiveness of an intervention, and building evidence-based guidelines [24]. Cohort identification can be accomplished by using phenotype definitions, which classify patients with a specific disease based on EHR data and can be manually developed by experts or machine learning. A phenotype definition shares some major features, such as logic, temporality, and the use of standard codes [44]. Furthermore, examples of data categories that are commonly used in phenotype definitions across institutions are “age, sex, race/ethnicity, height, weight, blood pressure, inpatient/outpatient diagnosis codes, laboratory tests, medications” [44]. On the other hand, there are some challenges with the cohort identification process that vary depending on the study type. The phenotyping process is more sophisticated than a simple code search [15]. Several factors can contribute to their complexity, including the used research methods and the presence of confounding factors. For example, when defining acute or less-defined phenotypes, one critical step is addressing confounding factors by using the matching of gender and age. These confounders are relatively easy to address, but others, such as co-diseases, might be more difficult. In a study completed by Castro et al. [45], they were not able to identify methods for matching controls in EHR data. Case–control studies may inherent some limitations in detecting comorbidities such as insufficient controls, identification of correct confounders, and case–control matching processes. Castro et al. [45] stated that their goal is to compare matching algorithms methods to identify clinically meaningful comorbidity associations. Literature-based comorbidity associations, derived by clinical experts from literature, are considered a reference standard to compare the performance of the matched controls. However, there were disagreements among gastroenterologist experts who compared the inflammatory bowel disease and disease associations found in Phenome-wide association studies (PheWAS) [46] disease groupings versus the associations found in the literature [45].

### Medical corpora for text mining

Many of the text mining applications require a corpus, a collection of text annotated by experts because these applications rely mostly on supervised machine learning methods. This is due to the challenges of recognizing terms as the example provided by Rodriguez-Esteban R [47] for: “the text ‘early progressive multifocal leukoencephalopathy’ could refer to any, or all, of these disease terms: ‘early progressive multifocal leukoencephalopathy’, ‘progressive multifocal leukoencephalopathy’, ‘multifocal leukoencephalopathy’, and ‘leukoencephalopathy’”. Such annotations based on expert knowledge can be used to train machines on, for example, recognizing biomedical terms in a text [47]. An annotated corpus requires experienced annotators, comprehensive guidelines, and large-scale high-quality corpora [48]. The manually annotated corpus can serve as a gold standard for building automated systems, e.g. statistical, machine learning, or rule-based [49]. Examples of annotated biological corpora are GENIA for annotating biological terms [50], BioCreative^1^ for annotating biological entities in literature e.g. genes and proteins [51], and BioNLP^2^ which is a collection of corpora, such as Colorado Richly Annotated Full-Text Corpus (CRAFT)^3^ and Protein Residue Corpora,^4^ for annotating biological entities. Another usage of an annotated corpus is to create a literature-based knowledgebase, such as MetaCore^5^ and BRENDA8^6^ for enzyme functional data [49]. However, these are mostly restricted to specific domains such as the biological domain which annotates information, such as gene names, protein names, and cellular location or events (e.g. protein–protein interaction) [49]. The availability of corpora in the medical domain is even more limited than in the biological domain. One of the major reasons is that the medical domain is confronted with data availability and ethical issues of using electronic medical records [49], including privacy and confidentiality and Health Insurance Portability and Accountability Act (HIPAA) regulations [52]. Examples of biomedical corpora are Text Corpus for Disease Names and Adverse Effects for annotating diseases and adverse effects entities [53], CLinical E-Science Framework (CLEF) for annotating medical entities and relations (e.g. drugs, indications, findings) in free texts of 20,000 cancer patient records [54], and Adverse Drug Effects (ADE) corpus^7^ for annotating ADEs entities [49]. None of the available corpora serves our needs for this task to annotate contextual cues of defining a phenotype in observational studies on sentence-level annotations from full texts, such as the presence of codes, laboratory tests, and type of data used.

An example of developing a corpus for phenotypes is PhenoCHF [55, 56], an annotated corpus by domain experts for phenotypic information relevant to Congestive Heart Failure (CHF) from literature and EHR. The PhenoCHF corpus data was derived from the i2b2 (the Informatics for Integrating Biology at the Bedside) discharge summaries dataset [57] and five full full-text articles retrieved from PubMed that covered the characteristics of CHF and renal failure. However, PhenoCHF focused only on one condition, CHF, and was built on a small corpus of only five full full-text articles. Furthermore, they did not annotate contextual cues for phenotyping case definitions. Intending to minimize human involvement, we realized that there is a lack of phenotyping tools [13] addressing or automating the extraction of existing definitions from the scientific literature.

There is no existing corpus that addressed the automatic identification of phenotype definitions on a sentence-level. In this study, our aim is to annotate a corpus that captures sentences with phenotypes and contextual cues and patterns of a phenotype definition that are presented in the literature. We believe that EHR-based studies will provide relevant information for defining phenotypes. An annotation guideline is developed and serves as a foundational approach for annotating phenotype definition information in the literature. Both the corpus and the guidelines are designed based on an extensive textual analysis of sentences to reflect phenotype definitions information and cues. Ten dimensions are proposed to annotate the corpus at the sentence-level. Furthermore, after identifying the presence or absence of the 10 dimensions, the level of evidence for each sentence was generated automatically using a rule-based approach to ensure consistency and accuracy of annotations. All sentences in the methodology section were extracted from full-text research articles. To the best of our knowledge, no annotated corpus is publicly available for annotating sentences with contextual cues of phenotype definitions from biomedical full-text articles for text mining purposes.

## Methods

The procedure of the corpus construction consists of document selection and sentence-level annotation [58]. The document selection started with the selection of phenotypes of interest that could assist in the searching for abstracts. After that, the collection of several abstracts was prepared, and full-text articles of selected abstracts were downloaded for the sentence-level annotation. For the sentence-level annotation, ten dimensions were proposed to annotate sentences with cues of phenotype definitions including biomedical terms and standard codes. Finally, conclusions were derived from each level of evidence in the sentences.

### Selection of phenotypes

Our research group was primarily interested in ADEs [59, 60]. Therefore, we identified our ADE phenotypes of interest based on our previous work of literature-based discovery [59, 60] that has identified DDIs due to interactions among five Cytochrome P450 (CYPs) enzymes, including CYP2C8, CYP2C9, CYP2C19, CYP2D6, and CYP3A. These CYPs have a significant role in drug metabolism leading to several DDIs [61, 62]. Furthermore, text-mining technology was used to extract DDI evidence and their corresponding ADEs from the biomedical literature. DDIs were identified with evidence in all types of DDI studies, including clinical pharmacodynamics (PD), clinical pharmacokinetics (PK), and in vitro PK studies [60]. Among those clinical PD abstracts with 986 drugs pairs, we explored ADEs from abstracts containing substrates of the five major metabolizing enzymes which are mentioned above. The drug-enzyme relationships were collected from the Flockhart table^8^ and FDA. As a result, a list of ADEs (*n* = 673) was used as the primary list of phenotypes. All the ADE terms for those substrates, which were matched with the preferred terms (PT), were found in the Medical Dictionary for Regulatory Activities Terminology (MedDRA) [63].

**Fig. 1:**
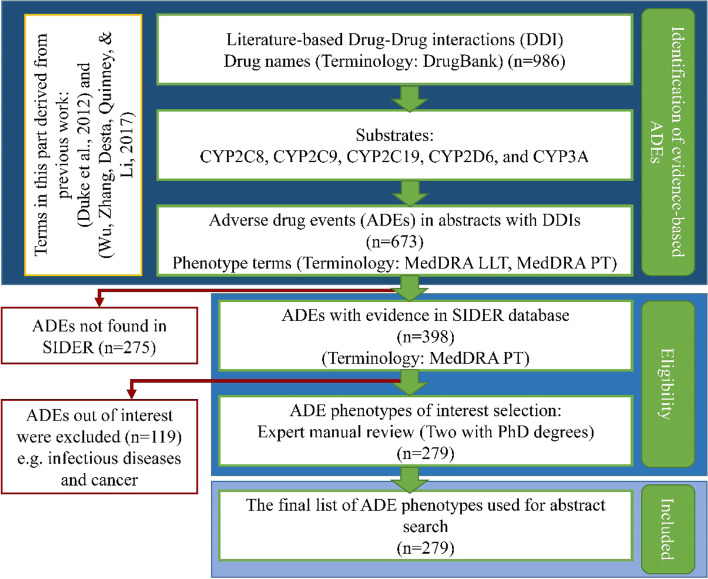
Flowchart of the selection process of the adverse drug event (ADE) phenotypes. The selection of the final list of ADE phenotypes started with the list literature-based discovery that has identified drug-drug interactions (DDIs) due to interactions among five Cytochrome P450 (CYPs) enzymes, including CYP2C8, CYP2C9, CYP2C19, CYP2D6, and CYP3A. This step was followed by the ADEs eligibility evaluation through evidence of drugs-ADEs linkage in the Side Effect Resource (SIDER) database and expert manual review to include the final list of ADE phenotypes of interest

To narrow down our phenotypes of interest, we identified ADEs that showed evidence of drugs-ADEs linkage in the Side Effect Resource (SIDER) database [64] and found that 398 ADEs were successfully linked to the side effects in the SIDER database. In the end, expert reviews were performed by two co-authors of this study, Lang Li, Ph.D. and Sara Quinney, Pharm.D., Ph.D., to finalize the list of phenotypes of interest. The experts excluded ADE terms that did not meet our lab research interests, such as terms related to infections and cancer. The final list of phenotypes of interest has 279 ADEs (Supplementary 1). Figure 1 shows the process of the selection of phenotypes.

### Abstracts and full texts collection and selection

To search the literature for observational studies, we consulted a medical librarian to assist in building search queries to ensure the highest coverage. A review study reported that due to the broad nature of phenotyping studies, it might be difficult to perform one search that is capable of capturing all EHR phenotyping studies [15]. Therefore, we collected our abstracts based on two search criteria:

First, we searched the PubMed database to identify observational studies of our phenotypes of interest. The searching query consisted of a phenotype of interest term (see Supplementary 1 for the list of ADE phenotypes of interest) combined with a set of keywords that were tested to retrieve relevant observational studies (see Table 1). We did not put restrictions on the year of publication and the search was performed in November 2017. The total number of retrieved abstracts without duplications was 1323 abstracts. One reviewer manually reviewed each abstract to select articles that met the inclusion criteria described in Table 1. Table 1 also shows the exclusion criteria that were applied to exclude abstracts. A total of 800 abstracts met our inclusion criteria. From the 800 abstracts, a subset of 57 abstracts was randomly selected for the full-text sentence-level annotation task (PMIDs in Supplementary 2).

**Table 1:** Abstract inclusion–exclusion criteria

**Searching Query**	**[A phenotype of interest term]**^a^ AND electronic health record (code OR codes OR algorithm^a^ or "case definition" OR "phenotyping" OR "case identification" OR claim OR administrative)
Inclusion Criteria	1. Abstracts should satisfy each of the following: English, full text available, and original research 2. The primary source of data is electronic health record (EHR) or electronic medical record (EMR). Some accepted terms: Registry, administrative data 3. The article should use observational data (population-based, surveillance, or cohort/cases) either retrospectively or prospectively 4. Clearly describe a case definition or algorithm according to any of the following criteria: coding algorithms (SNOMED, ICD9/10, CPT, LOINC, RxNorm, UMLS, READ), laboratory, natural language processing (NLP), or inclusion and exclusion criteria
Exclusion Criteria	1. Review articles 2. Non-human studies 3. Nurses/practitioners as the primary population of the study 4. Not real-world data: e.g. simulation data 5. Tools, systems, or reporting systems that do not address phenotyping or description phenotype definition

^a^See Supplementary 1 for the list of ADE phenotypes of interest

Second, we used abstracts from a previous search that was performed by two reviewers. The used search queries were more generalized such as “electronic health record AND myopathy” (All queries are presented in Supplementary 3). However, the disadvantage of these queries was that they generated a large number of abstracts that were time-consuming and labor-intensive to review all of them. The reviewers collected some relevant abstracts from these search queries. From these collected abstracts, we randomly selected 29 abstracts. The query searches with PMIDs are shown in Supplementary 3.

With this, the total number of abstracts derived from the two search criteria was 86 abstracts. We achieved this number based on our goal to reach around 4000 sentences from the method sections. We downloaded their full texts and tokenized them into sentences using a package called ‘Perl::Tokenizer’ as preparation for the annotation process. In addition, we manually fixed sentences that were tokenized improperly. After that, we extracted sentences within the method sections.

### Corpus construction

The annotation guidelines were developed based on textual analysis of the contextual cues in sentences with a phenotype definition that was inspired by major data modalities of phenotype definitions used in PheKB [9]. We performed sentence-level annotations with three major categories for each sentence, which were: inclusion, intermediate, and exclusion. The sentence-level annotations’ categories were derived based on the availability of ten dimensions that are shown in Table 2 with their descriptions and examples. The detailed annotation guidelines are available in Supplementary 4.

**Table 2:** Sentence-level annotation’s categories, dimensions, and sub-dimensions

Category, dimension, and sub-dimension	Description	Examples (Sentences)
**1. Inclusions category (*n*** **= 5)**		
1.1. Biomedical & Procedure	Evidence of defining a phenotype when biomedical and procedure entities co-occur with phenotype definition cues	“dyslipidemia was defined as total cholesterol greater than 220 mg/dl…” (PMID:20819866)
1.2. Standard Codes	Evidence of using standard terminologies that are commonly used in a clinical setting. Examples of these standard coding classifications and/or terminologies are ICD-9/10, SNOMED CT, and CPT codes	“a primary or any secondary discharge diagnosis (International Classification of Diseases, Ninth Revision, Clinical Modification [ICD-9-CM] code) of myoglobinuria (791.3)”. (PMID:15572716)
1.3. Medications	Evidence of the use of medication for defining a phenotype	“The use of a lipid-lowering medication”. (PMID:20819866)
1.4. Laboratories	Evidence of using quantitative values reflecting clinical measurable values (i.e. laboratory tests values, vital values, procedures, clinical)	“Dyslipidemia was defined as total cholesterol greater than 220 mg/dl”. (PMID:20819866)
1.5. Use of Natural Language Processing (NLP)	Evidence of NLP uses accompanied with any of the following entities: biomedical, procedure, and/or medications	“The algorithm uses nonnegated terms indicative of HF: cardiomyopathy, heart failure, congestive heart failure, pulmonary edema, decompensated heart failure, volume overload, and fluid overload”. (PMID:17567225)
**2. Intermediate category (*n*** ** = 2)**		
1.6. Data sources	Evidence of information relevant to data sources used in the study or the phenotype definition. Some examples when describing a database used, clinical data, and/o electronic health records (EHR)	“Computerized medical and pharmacy records were reviewed”. (PMID:11388131)
1.7. Study design or IRB	Evidence of information about study design or the IRB. For example, evidence of the method used as “Gold standard”	“STUDY DESIGN: Retrospective chart review”. (PMID:11388131)
**3. Exclusion category (*n*** ** = 3)**		
1.8. Exclusion 1– Irrelative evidence: 1.8.1. Location 1.8.2. Ethical 1.8.3. Financial 1.8.4. Patient direct contact 1.8.5. Provider or researchers (excluding patients) 1.8.6. Performance 1.8.7. Quality of Care	Evidence of information about other study methodological details that are not supportive for defining a phenotype directly	“All patients were members of the managed care system and incurred a significant financial advantage from having their prescriptions filled within the system”. (PMID:16765240)
1.9. Exclusion 2- Computational and statistical evidence: 1.9.1. Alerts 1.9.2. Software 1.9.3. Statistics	Evidence of computational or statistical information that is not supported for phenotype definitions	“We used logistic regression models with generalized estimating equations to adjust for race, year, race x year interactions, age, and sex”. (PMID:16567608)
1.10. Exclusion 3- Insufficient evidence: 1.10.1. Insufficient evidence	Sentences that do not show any evidence in any of the nine dimensions	“Fallon is offered by about 3,500 employers”. (PMID:12952547)

**Fig. 2:**
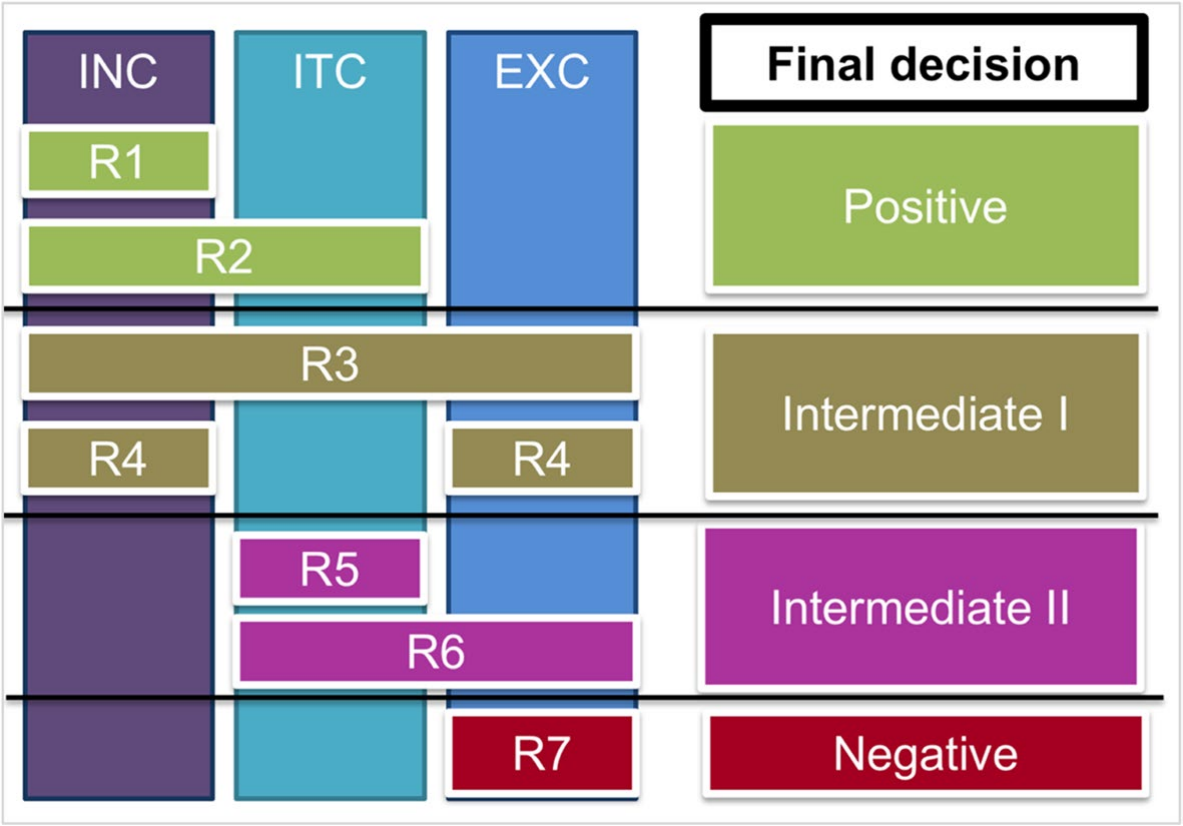
Level of evidence to build the final decision (Rule-based final decisions). Inclusion conclusion (INC); Intermediate conclusion (ITC); Exclusion conclusion (EXC). Rule 1 (R1) and Rule (R2)—Strong evidence of a phenotype definition; Rule 3 (R3) and Rule 4 (R4)—Strong intermediate evidence of a phenotype definition; Rule 5 (R5) and Rule 6 (R6)—Weak intermediate evidence of a phenotype definition; and Rule 7 (R7)—No evidence of a phenotype definition

Within the annotation construction, the inclusion category contained sentences that showed evidence of at least one of the dimensions that characterized a phenotype definition (Table 2). We identified five dimensions for the inclusion category, which were “Biomedical & Procedure”, “Standard codes”, “Medications”, “Laboratories”, and “Use of NLP”. The proposed dimensions were represented either as keywords or more complex such as events where co-occurrence of more than one keyword occurs. For example, the “Standard codes” dimension was represented by the presence of any keyword relative to “Standard codes”, such as ICD-9, Systemized Nomenclature of Medicine – Clinical Terms (SNOMED CT) [65], or a diagnostic code. On the other hand, “Biomedical & Procedure”, “Medications”, “Laboratories”, and “Use of NLP” required an event presence such as the co-occurrence of two keywords that were identified for each dimension. Sentences were categorized as positive for the inclusion category if they showed evidence of any of the five dimensions (Table 2), which satisfied the inclusion conclusion (INC) criteria (See Fig. 2 and Table 3).

**Table 3:** Level of evidence of a sentence with a phenotype definition (Rule-based final decisions)

Rule	Rule description		Level of evidence	Final Decision	Number of Sentences (%)
R1	If INC = True and ITC = False and EXC = False	The sentence shows **strong evidence** of a phenotype definition		Positive	1222 (30.77%)
R2	If INC = True and ITC = True and EXC = False	The sentence shows **strong evidence** of a phenotype definition			
R3	If INC = True and ITC = True and EXC = True	The sentence shows **strong intermediate evidence** of a phenotype definition due to the presence of any of the Exclusion criteria		Intermediate I	701 (17.65%)
R4	If INC = True and ITC = False and EXC = True	The sentence shows **strong intermediate evidence** of a phenotype definition due to the presence of any of the Exclusion criteria			
R5	If INC = False and ITC = True and EXC = False	The sentence shows **weak intermediate evidence** of a phenotype definition due to the absence of any of the Inclusion criteria, but the presence of any of the intermediate criteria		Intermediate II	914 (23.01%)
R6	If INC = False and ITC = True and EXC = True	The sentence shows **weak intermediate evidence** of a phenotype definition due to the absence of any of the Inclusion criteria, but the presence of any of the intermediate criteria			
R7	If INC = False and ITC = False and EXC = True	The sentence shows **no evidence** of a phenotype definition		Negative	1134 (28.55%)

*INC* Inclusion conclusion, *ITC* Intermediate conclusion, *EXC* Exclusion conclusion

Secondly, the intermediate category included sentences that did not show direct evidence of a phenotype definition, but could assist by providing supporting evidence for phenotyping. We identified two dimensions for the intermediate category, which were “Data sources” and “Study design or Institutional Review Board (IRB)”. Since different studies have shown varying research questions and designs [24], the intermediate category could assist in capturing data sources information that matched the study’s goals. A sentence was categorized as positive for the intermediate category if it showed evidence of any of the two dimensions (Table 2), which we called intermediate conclusion (ITC) (Fig. 2 and Table 3).

Thirdly, the exclusion category included sentences that were out of the scope of a phenotype definition or phenotyping. A sentence was categorized as positive for the exclusion category if it showed evidence of any of the three dimensions (Table 2), which we called exclusion conclusion (EXC) is true (Fig. 2 and Table 3).

Finally, the final decision is the overall sentence-level of evidence derived from INC, ITC, and EXC (Fig. 2 and Table 3). We note that some sentences can have evidence of more than one dimension which determines final sentence-level conclusions (INC, INT, EXC) in Table 3. We used a rule-based approach to produce four final sentence-level decisions, which are “Positive”, “Intermediate I”, “Intermediate II”, and “Negative”. The goal was to create accumulative evidence in each sentence based on the presence of any of the three conclusions (INC, ITC, EXC). This helped to ensure consistency, accuracy, and quality of the annotations. Table 3 and Fig. 2 show the criteria of the seven rules (R1, R2, R3, R4, R5, R6, and R7). We combined R8 final decision where all the three conclusions (INC, ITC, EXC) were false with R7 indicating negative evidence.

### Annotation process

**Fig. 3:**
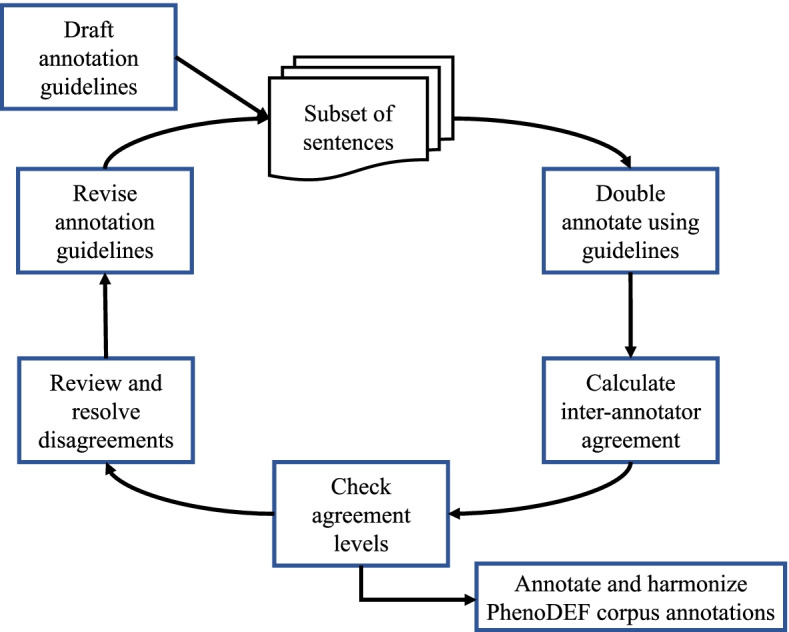
The annotation process of developing the annotation guidelines and the final annotation. The annotation task was an iterative process that started with a subset of sentences. Two annotators annotated the subset of sentences, calculated the inter-annotator agreement, and checked the agreements. If disagreements existed, a third annotator resolved the disagreements between the two annotators. Then, the annotators revised and drafted the annotation guidelines. This was an iterative process until no further disagreements existed

To produce a high-quality corpus, it is recommended that the corpus is annotated by more than one annotator [66]. Here, two annotators with a biomedical informatics background (SB, HW) carried out the annotation process. Both annotators have degrees in biomedical informatics, are familiar with the medical standard terminologies, and are familiar with text mining. We designed the annotation guidelines iteratively through several meetings and manual analysis of textual patterns of a phenotype definition. When both annotators were satisfied with the final version of the annotation guidelines, they started the annotation of the corpus. For each dimension of the ten dimensions (Table 2), if the dimension was present, the annotator annotated it as 1, otherwise, it was annotated as 0. The development of annotation guidelines was critical to ensure the consistency and quality of the annotations. The process usually starts with a draft, and can then be refined iteratively until the final draft is accomplished [49]. During the guideline’s development process, subsets of the corpus were annotated until the annotators were satisfied with the guidelines. After that, the full corpus was annotated. The process is shown in Fig. 3 which was inspired by Gurulingappa et al. annotation task workflow [49].

After finalizing the guidelines, both annotators annotated all sentences of the corpus following the final proposed annotation guidelines. The annotation process was divided into several rounds starting from the annotation of a subset of sentences 400 (first round). After that, the number of sentences for each round was 1000, 1300, and 2700. After each annotation round, there were “consensus sessions” that each took around 1 h to 4 h where annotators discussed and resolved any disagreements. Moreover, a third annotator (LL) addressed disagreements in annotations between annotators if they did not achieve a consensus. The goal was to identify areas of disagreement as well as areas to build our gold standard.

### Inter-annotator agreement (IAA)

The inter-annotator agreement is to assess the reliability of the annotations. There are several benefits of the manual annotation by multiple people, such as generating correct annotations, validating and improving the scheme guidelines, resolving ambiguities in data, and evaluating valid interpretations [66]. Further, the written annotation guidelines scheme help in generating consistent and reproducible annotations [66]. Therefore, to measure the agreement between annotators, we used three measures of agreement: percent agreement, overall percent agreement [67], and Cohen’s kappa [68]. These measures vary in their approaches, but they all aim at producing the best possible reliable and correct annotations as there is no reference for the annotation of some of the sources [66]. The percent agreement and Cohen’s kappa [68] were calculated for each dimension using R packages (‘irr’^9^ for percent agreement and ‘fmsb’^10^ for kappa). For example, if the two annotators annotate a dimension as 1, it means an agreement. On the other hand, if one annotator annotates a dimension as 1 and the other as 0, it means disagreement. The overall percent agreement [67] was calculated over the ten (10) dimensions on a sentence-level (Table 2) as the following:

$$\mathrm{Overall}\;\mathrm{sentence}\;\mathrm{level}\;\mathrm{agreement}=\frac{\left(Number\;of\;Sentences\times10\right)-Number\;of\;disgreement}{(Number\;of\;Sentences\;\times10)}\times100$$

## Results

### Corpus description

PubTator^11^ is a web-based tool for annotating biomedical entities, including diseases, genes, mutations, and chemicals [69]. We uploaded our PMID list (*n* = 86) and run the annotation analysis. Table 4 presents the results from PubTator for the disease terms that were found in more than one abstract. Disease terms that appeared in single abstracts and terms for other entities (genes, mutations, and chemicals) are shown in Supplementary 5.

**Table 4:** Phenotypes that appeared in more than one abstract in our corpus

Term	Number of abstracts
Diabetes	16
Hypertension	11
Diabetes mellitus	8
Heart failure	7
Asthma	3
Bleeding	3
Cancer	3
Coronary heart disease	3
Diabetic	3
Hypertensive	3
Obesity	3
Osteoarthritis	3
Pneumonia	3
Type 2 diabetes	3
Acute renal failure	2
Allergies	2
Death	2
Dementia	2
Gout	2
Myocardial infarction	2
Pulmonary embolism	2
Rhabdomyolysis	2
Rheumatoid arthritis	2
Right bundle branch block	2
Sepsis	2
Stroke	2

We found the following study design terms as they appeared in the text in our corpus, including observational study, longitudinal study, cohort study (retrospective cohort, prospective cohort, nonrandomized retrospective cohort study), case–control study, retrospective study (retrospective cohort, nonexperimental retrospective, non-randomized retrospective cohort study, retrospective validation), cross-sectional study, comparative study, descriptive study, validation study, prospective study (prospective cohort study), genome-wide association study, epidemiology and/or surveillance study, and follow-up study.

We annotated the corpus using our annotation guidelines with three categories and ten dimensions (Table 2), which are the Inclusion category (“Biomedical & Procedure”, “Standard codes”, “Medications”, “Laboratories”, “Use of NLP”), Intermediate category (“Data sources”, “Study design and/or IRB”), and Exclusion category (“Irrelative evidence”, “Computational and statistical evidence”, “Insufficient evidence”). The total number of sentences in this corpus was 3971 sentences that were extracted from 86 full texts methods sections. Table 5 shows the number of annotated sentences for each category and dimension. “Biomedical & Procedure” dimension showed the highest number of annotated sentences with around 1449 (36.5%). “Data sources” and “EXC2 – Computational and statistical evidence” were both over thousand annotated sentences with 1370 (34.5%) and 1314 (33.1%), respectively. The number of annotated sentences for “Medications”, “Standard codes”, and “Laboratories” dimensions from the inclusion category was 593 (14.9%), 385 (9.7%), and 246 (6.2%). The number of annotated sentences for the “Use of NLP” dimension was the lowest with 49 (1.2%).

**Table 5:** Corpus description and inter-annotator agreement

Category	Number of sentences (%) per category	Dimension	Number of sentences (%) per dimension	Percent	Kappa	Kappa 95% CI
Inclusion	1923 out of 3971 (48.4%)	Biomedical & Procedure	1449 (36.5%)	95.00%	88.96%	0.87—0.90
		Standard codes	385 (9.7%)	99.47%	97.01%	0.95—0.98
		Medications	593 (14.9%)	99.09%	96.44%	0.95—0.97
		Laboratories	246 (6.2%)	99.70%	97.42%	0.95—0.98
		Use of Natural Language Processing (NLP)	49 (1.2%)	99.65%	83.54%	0.74—0.92
Intermediate	1851 out of 3971 (46.6%)	Data sources	1370 (34.5%)	96.71%	92.59%	0.91—0.93
		Study design and/or Institutional Review Board (IRB)	780 (19.6%)	98.00%	93.56%	0.92—0.94
Exclusion	2273 out of 3971 (57.3%)	Irrelative evidence	733 (18.4%)	97.27%	91.05%	0.89—0.92
		Computational and statistical evidence	1314 (33.1%)	96.84%	92.83%	0.91—0.94
		Insufficient evidence	359 (9.0%)	95.96%	78.72%	0.75—0.82

Table 3 (see 5 section) shows the rule-based final decisions which are “Positive”, “Intermediate I”, “Intermediate II”, and “Negative”. The positive indicated the highest level of evidence of defining a phenotype while the negative indicated no evidence of defining a phenotype. The number of sentences with “Positive” is 1222 (30.77%). “Intermediate I” is the sentence that showed strong intermediate evidence were 701 (17.65%) sentences of the corpus. “Intermediate II” are the sentences that showed weak intermediate evidence were 914 (23.01%) sentences of the corpus. Finally, the number of negative sentences represented in our corpus was 1134 (28.55%) sentences.

### Inter-annotator agreement

For inter-annotator agreement, the calculations were based on the annotation of each dimension (Tables 2 and 5). We used the *overall sentence-level percent agreement* (inspired by Wilbur et al. [67]), *percent agreement*, and *Kappa*. The *overall sentence-level percent agreement* was high at 97.8%. The *percent agreement* and *kappa* measures results are shown in Table 5.

Generally, all dimensions showed high agreement on both *percent agreement* and *kappa*. For the dimensions of the inclusion category, the “Biomedical & Procedure” showed 95% *percent agreement*, and almost perfect *kappa* with 88.96%. For the “Standard codes”, “Medications”, and “Laboratories” dimensions, they all showed over 99% *percent agreement* and over 96% *kappa*. For the “Use of NLP” dimension, it showed over 99% *percent agreement* and 83.54% *kappa*. For the dimensions of the intermediate category, they showed high agreement on *percent agreement* with over 96%, and *kappa* with over 92%. Finally, for the dimensions of the exclusion category, both “EXC1 – Irrelative evidence” and “EXC2 – Computational and statistical evidence” showed high agreement on *percent agreement* with 97.27% and 96.84%, and *kappa* with 91.05% and 92.83%, respectively. The “EXC 3 – Insufficient evidence” dimension showed high *percent agreement* (95.96%) and substantial *kappa* (78.72%).

### Error analysis

We performed an error analysis on sentences where annotators had disagreements. Table 6 provides some common errors that led to some of the disagreements between the annotators with examples. For example, we found that recognizing abbreviated terms was slightly challenging and it appeared problematic in seven dimensions. It can be hard to determine if an abbreviated term is a biomedical, procedure, medication, or standard code, such as the abbreviated term “ICD” which might mean “Implantable Cardiac Defibrillators” or “International Classification of Diseases”. Furthermore, there was an ambiguity in some of the terms that the same term has more than one meaning, such as “mean” which can refer to the statistical measure “mean” or the word “mean”.

**Table 6:** Error analysis of the annotation with disagreements

Error	Dimension	Examples (Sentences)
**Abbreviated terms**	Biomedical & Procedure	"Events that occurred during follow-up were identified from hospitalization records, and ARIC and CHS study" (PMID25104519)
	Standard codes	"Finally, the Apollo Data Repository provided data for ICDs" (PMID26961369)
	Medications	" ‘‘common’’ side effects, e.g. headache, to judge the relevance of side effects associated with AZA”. (PMID24177317)
	Use of NLP	"From this cohort, we identified 15,761 patients with HPI that was processed through a natural language processing algorithm…” (PMID25567824)
	Data	“Cohort with HPI data” (PMID25567824)
	EXC1 – irrelevant evidence	"190 patients completed the SCID assessment"(PMID25827034)
	EXC2 – Computational and statistical evidence	"The MCMC method" (PMID21931496)
**Missed keywords or criteria**	Use of NLP	"The algorithm uses non-negated terms indicative of HF" (PMID17567225)
	Data	"If data on weight and height were available” (PMID21862746)
	EXC1 – irrelevant evidence (financial)	"until termination of insurance coverage”. (PMID12952547)
	EXC1 – irrelevant evidence (ethical)	"To protect patient confidentiality, all personal identifiers are deleted” (PMID21051745)
	EXC1 – irrelevant evidence (location of the study)	"We randomly sampled outpatient clinical encounters from October 1, 2003 through March 31, 2004 at VA Maryland (VAMHCS) and at VA Salt Lake City (VASLCHCS) Health Care systems”. (PMID20976281)
	EXC2 – Computational and statistical evidence	"Characteristics were measured during the one-year baseline period (i.e., before time zero)”. (PMID20112435)
**Without co-occurrence with a biomedical, procedure, or medication terms**	Use of NLP	"Humedica derives NLP items from text entries that correspond primarily to terms in two large dictionaries, SNOMED and MedDRA" (PMID26725697)
	Data	"If the first record for a woman was either …" (PMID22071529)
**Term ambiguity**	Biomedical & procedures events	"Only acute conditions occurring during the first 24 h of hospital admission were considered”. (PMID24734124)
	Study design or IRB	"The nucleotide reference for this allele is guanine. 4″. (PMID26221186)
	EXC2 – Computational and statistical evidence	"More points mean a higher risk of hyperkalemia”. (PMID20112435)
**Neither biomedical nor procedure (e.g. Social status)**	Biomedical & Procedure	"We created a binary variable for marital status, where “single” included those patients classified as divorced, single, widowed, or separated”. (PMID25091637)
**A not clear statement of using standard codes**	Standard codes	"Outcomes were evaluated by administratively coded data” (PMID26370823)
**Assigning terms as biomedical & procedure vs. medications (e.g. substances)**	Biomedical & Procedure/Medications	"The most recent fasting lipid profile in patients with dyslipidemia and glycosylated hemoglobin level in patients with diabetes” (PMID11388131)
**Spelling and short forms**	Medications	"Asthma meds refilled regularly”. (PMID12952547)
**Without co-occurrence with supportive definition evidence**	Biomedical & Procedure/Medications	"reports KD = 9100 for bupropion and KD > 10 000 for mirtazapine (vs 200 for nefazodone)”. (PMID22466034)
**“More than or less than” value, but not directly relevant to phenotyping**	Clinical measurable values	" ≥ 2 years of observation before the period of interest; *n* = 50″. (PMID23449283)
**New keywords for the dimension**	EXC2 – Computational and statistical evidence	Examples of new keywords describing “EXC2” are: risk score, inter-rater variability, custom-designed data entry template, predictor variable, Tukey multiple comparison test, Web-accessible, teleconferences, propensity-matched, machine-implementable rule, Illumina Omni1_- QUAD, Illumina 660 W, TaqMan, Illumina 660-Quad, and Illumina

## Discussion

In this work, our goal was to develop an annotation approach and an annotated corpus that can support future text-mining tasks such as the literature-based discovery of phenotyping case definitions. In terms of selection of phenotypes, we chose to select a set of phenotypes based on our group research interests, which were mostly ADEs (*n* = 279). We utilized these phenotypes to search the literature for abstracts and we included 86 abstracts to build the sentence-level corpus from their full texts’ methods sections. Annotation approaches were based on evaluating the presence of our proposed ten dimensions in a sentence (Table 2) and the final decisions were derived based on a set of seven rules (Table 3). Our focus in annotating the corpus is to develop a generalized approach to capture contextual features of phenotyping rather than focusing on specific entities. The two annotators worked on developing the annotation guidelines iteratively; after finalizing the guidelines, the whole corpus was annotated. For inter-annotator agreement, we used three measures for evaluation: *overall sentence percent agreement* (inspired by Wilbur et al. [67]), *percent*, and *kappa agreement*. Overall, the results for the inter-annotator agreement were high and the *overall sentence-level percent agreement* was high at 97.8%. One observation with the “EXC 3 – Insufficient evidence” dimension showed “substantial agreement” (see Table 2 for interpretation of Kappa in [70]) that was the lowest kappa score among all dimensions. This dimension indicates sentences with a lack of evidence in any of the other nine dimensions. Overall, we annotated 3971 sentences extracted from methods sections of 86 articles and the inter-annotator agreement showed that the annotations and guidelines are valid.

### Sentence-level annotation and dimensions selection

Our decision in this work is to focus on sentence-level annotations rather than entity-level annotations. There are several reasons for this decision. First, we believe that a phenotype definition is best represented as full sentences rather than single concepts or terms. Entity-level annotations can be accomplished in future steps with the goal of text summarization and information extraction. Second, we aimed to utilize a generalizable approach that serves as a foundational basis for annotating a phenotype definition. The selection of ten proposed dimensions (Table 2) was based on identifying phenotype definition contextual cues that were observed in the published literature [9, 13, 24, 41] as well as during our manual annotation process (Fig. 2). Third, based on our analysis, contextual cues of a phenotype definition are not only reliant only on biomedical terms, but also it can be extended to other cues, such as “defined”, “inclusion criteria”, “exclusion criteria”, and “eligibility”. To our knowledge, contextual cues and patterns of phenotype definitions in the literature on a sentence-level were not studied previously.

### Error analysis

Recognizing abbreviated terms was slightly challenging and it appeared problematic in seven dimensions. Some of the disagreements were resolved by returning to the full-text article. For terms with ambiguity, understanding the context around the text was necessary and helped in addressing this problem. In addition, we observed that natural human error generated some disagreements during the annotation process. For example, one of the annotators missed some keywords for some dimensions which we identified during the consensus sessions. Such mistakes were not intentionally made. Overall, annotating phenotype definitions’ events e.g. a co-occurrence of more than one keyword is challenging because they require the presence of more than one pattern.

### Study limitations and future work

This work does not stand without limitations. Annotating a larger number of articles might generate more contextual patterns of a phenotype definition in EHR-based studies. However, we also believe that we have comprehensive coverage for several study types of studies. With the multi-study coverage, we believe that our corpus was sufficient to capture a wide range of contextual cues representing a phenotyping case definition in the biomedical literature. Furthermore, we believe that our approach can be generalizable and scalable to other phenotypes because our intention was on the phenotype definitions contextual cues and patterns, and we did not limit the sentence-level annotations to the ADE phenotypes.

The manual corpus annotation is time-consuming and labor-intensive. Only two annotators annotated the corpus; therefore, we tested the annotations with more than one measurement of agreement (overall percent, percent, and kappa). Both annotators were familiar with biomedical informatics concepts and text mining approaches, but we note that some were more challenging than others. The results of inter-annotator agreement showed high agreement indicating reliable annotations and guidelines. Generally, more annotators with clinical expertise could assist more during the task of annotations. In addition, text mining methods, such as named-entity recognition (NER) which is a subtask of information extraction [71], can be used to automatically recognize entities or phenotypes within the phenotype definitions sentences can also improve the annotation process and decrease the time of annotation. For example, NER can utilize existing medical terminologies and classifications, such as Unified Medical Language System (UMLS) [72, 73], to recognize ADE, biomedical, procedure, social descriptors, and other phenotype categories. For example, a list of ADE phenotypes of interest can be mapped to all synonyms to be used to recognize all ADE entities within a text. However, the entity-level annotation was out of the scope of this work.

For the “Use of NLP” dimension, the number of sentences was comparably lower than the number of sentences in other dimensions. However, since we decided to only annotate the presence or absence of NLP in a sentence to use it as a part of a phenotype definition, going beyond this scope might complicate the annotation task. For instance, detailed annotations of NLP methodology might be needed which was out of the scope. In addition, our aim in this work is to establish a foundational approach for annotating phenotypes definitions published in the literature. Future work can focus on annotating NLP methods contained within a phenotype definition.

To date, PheKB [9] library provides around 50 definitions only for some phenotypes. A study of best practices for phenotyping of adverse events found that the re-utilization of existing definitions is crucial [74]. This only works for case definitions that have been already published in the literature. Therefore, this work aimed to support the reusability of published definitions [7] by analyzing their contextual cues. Specifically, for using case definitions to establish EHR-based research, such as drug safety surveillance. Availability of the phenotype definitions can also assist in the validation of them in several institutions to ensure cohort consistency [75]. The ten dimensions in our annotation guidelines provide a foundational understanding of the basic contextual cues that represent a phenotyping case definition in the literature. Therefore, we believe that this corpus can serve as a baseline for developing either automatic or manual approaches to annotate a larger corpus size and advancing our proposed guidelines. Furthermore, our main aim in developing this corpus is to use it for text-mining applications to automate the mining of phenotype definitions published in the literature. For example, future work can train word vectors on the abstract-level and full-text sentence-level.

## Conclusions

In conclusion, clinical research, such as drug discovery, is moving towards the use of EHRs and electronic phenotyping where phenotype definitions are needed. We believe that literature provides an important source of data for mining phenotype definitions’ information. The corpus and annotation guidelines can serve as a foundational informatics approach for annotating and mining literature-based phenotype definitions. Ten dimensions on a sentence-level were proposed characterizing major contextual patterns and cues of a phenotype definition in published literature. This is a step towards research to advance leveraging of phenotype definitions from literature to support EHR-based phenotyping studies.

## Supplementary Information

**Additional file 1:** **Supplementary 1.** Phenotypes of interest: list of 279 potential adverse drug events (ADEs). **Supplementary 2.** PMIDs selected by searching criteria explained in (Table 1 Abstract Inclusion-Exclusion criteria). **Supplementary 3.** Abstracts selected by using the other searching criteria (Not Table 1 Abstract Inclusion-Exclusion criteria). **Supplementary 4.** Annotation guidelines to annotate a phenotype definition in the literature. **Supplementary 5.** Entities and terms in the 86 abstracts using PubTator annotation tool.

## Data Availability

The annotated corpus is available through an Open Science Framework (OSF) project page at https://osf.io/56fua/?view_only=d38be18542c740b6aae96f5b53c2eda0.

## Abbreviations

ACE-I: Angiotensin-converting enzyme inhibitor
ADE: Adverse drug event
CHF: Chronic Heart Failure
CRS: Chronic rhinosinusitis
CLEF: CLinical E-Science Framework
CRAFT: Colorado Richly Annotated Full-Text Corpus
CT: Computed tomography
CPT: Current Procedural Terminology
CYP: Cytochrome P450
DDI: Drug-drug interaction
EHR: Electronic health record
eMERGE: Electronic Medical Records & Genomics
EXC: Exclusion conclusion
HIPAA: Health Insurance Portability and Accountability Act
INC: Inclusion conclusion
IRB: Institutional Review Board
ITC: Intermediate conclusion
ICD-9: International Classification of Diseases, Ninth Revision
MedDRA: Medical Dictionary for Regulatory Activities Terminology
NLP: Natural language processing
PD: Pharmacodynamics
PheWAS: Phenome-wide association studies
PheKB: Phenotype KnowledgeBase
PK: Pharmacokinetics
PT: Preferred terms
SIDER: Side Effect Resource
SNOMED CT: Systemized Nomenclature of Medicine – Clinical Terms
SHARP: Strategic Health IT Advanced Research Projects
FDA: Food and Drug Administration
UMLS: Unified Medical Language System

## Footnotes

1 http://www.biocreative.org/news/corpora/biocreative-iii-corpus/

2 http://bionlp-corpora.sourceforge.net/

3 http://bionlp-corpora.sourceforge.net/CRAFT/index.shtml

4 http://bionlp-corpora.sourceforge.net/proteinresidue/index.shtml

5 http://www.genego.com/metacore.php

6 http://www.brenda-enzymes.org/

7 https://sites.google.com/site/adecorpus/home/document

8 https://drug-interactions.medicine.iu.edu/Main-Table.aspx

9 https://cran.r-project.org/web/packages/irr/irr.pdf

10 https://cran.r-project.org/web/packages/fmsb/fmsb.pdf

11 https://www.ncbi.nlm.nih.gov/research/pubtator/
