# Correction: PhenoDEF: a corpus for annotating sentences with information of phenotype definitions in biomedical literature

**DOI:** 10.1186/s13326-022-00275-3

**Published:** 2022-07-20

**Authors:** Samar Binkheder, Heng-Yi Wu, Sara K. Quinney, Shijun Zhang, Md. Muntasir Zitu, Chien-Wei Chiang, Lei Wang, Josette Jones, Lang Li

**Affiliations:** 1 Department of Biohealth Informatics, Indiana University School of Informatics and Computing, Indianapolis, IN USA; 2 Medical Informatics Unit, Department of Medical Education, College of Medicine, King Saud University, Riyadh, Saudi Arabia; 3 Development Science Informatics, Genentech, South San Francisco, CA USA; 4 Department of Obstetrics and Gynecology, Indiana University School of Medicine, Indianapolis, IN USA; 5 Department of Biomedical Informatics, College of Medicine, The Ohio State University, Columbus, OH USA; 6 250 Lincoln Tower, 1800 Cannon Drive, Columbus, OH 43210 USA

**Correction: J Biomed Semantics 13, 17 (2022)**

**https://doi.org/10.1186/s13326-022-00272-6**

Following publication of the original article [1], we have been notified that the numbering of column 1 in Table 2 is incorrect. It should be as follows:

2. Intermediate category (n =2)

2.1. Data sources

2.2. Study design or IRB

3. Exclusion category (n =3)

3.1. Exclusion 1– Irrelative evidence:

3.1.1. Location

3.1.2. Ethical

3.1.3. Financial

3.1.4. Patient direct contact

3.1.5. Provider or researchers (excluding patients)

3.1.6. Performance

3.1.7. Quality of Care

3.2. Exclusion 2- Computational and statistical evidence:

3.2.1. Alerts

3.2.3. Software

3.2.4. Statistics

3.3. Exclusion 3- Insufficient evidence:

3.3.1. Insufficient evidence
