# Supplementary material for: PhenoDEF: a corpus for annotating sentences with information of phenotype definitions in biomedical literature
Source: J Biomed Semantics. 2022 Jun 11;13:17. doi: 10.1186/s13326-022-00272-6 (PMC9188713; doi:10.1186/s13326-022-00272-6)
Supplement: Supplementary file 1 — Additional file 1: Supplementary 1. Phenotypes of interest: list of 279 potential adverse drug events (ADEs). Supplementary 2. PMIDs selected by searching criteria explained in (Table 1 Abstract Inclusion-Exclusion criteria). Supplementary 3. Abstracts selected by using the other searching criteria (Not Table 1 Abstract Inclusion-Exclusion criteria). Supplementary 4. Annotation guidelines to annotate a phenotype definition in the literature. Supplementary 5. Entities and terms in the 86 abstracts using PubTator annotation tool. [file 13326_2022_272_MOESM1_ESM.docx]

SUPPLEMENTARY MATERIAL

Supplementary 1 Phenotypes of interest: list of 279 potential adverse drug events (ADEs)

| **ADE-related phenotypes (1-150)** | **MedDRA**  **PT** | **ADE-related phenotypes (151-279)** | **MedDRA**  **PT** |
| --- | --- | --- | --- |
| Abscess | 10000269 | Insomnia | 10022437 |
| Acne | 10000496 | Irritability | 10022998 |
| Acute coronary syndrome | 10051592 | Ischaemia | 10061255 |
| Affect lability | 10054196 | Ischaemic stroke | 10061256 |
| Aggression | 10001488 | Jaundice | 10023126 |
| Agitation | 10001497 | Lethargy | 10024264 |
| Akathisia | 10001540 | Leukocytosis | 10024378 |
| Alopecia | 10001760 | Leukocyturia | 10050791 |
| Anaemia | 10002034 | Leukopenia | 10024384 |
| Aneurysm | 10002329 | Lipoatrophy | 10024604 |
| Angina pectoris | 10002383 | Lipodystrophy acquired | 10049287 |
| Anxiety | 10002855 | Liver disorder | 10024670 |
| Anxiety disorder | 10057666 | Liver injury | 10067125 |
| Arrhythmia | 10003119 | Lung disorder | 10025082 |
| Arteriosclerosis | 10003210 | Lymphocele | 10048642 |
| Arthritis | 10003246 | Lymphoproliferative disorder | 10061232 |
| Asthenia | 10003549 | Malaise | 10025482 |
| Asthma | 10003553 | Mania | 10026749 |
| Ataxia | 10003591 | Menorrhagia | 10027313 |
| Atrial fibrillation | 10003658 | Methaemoglobinaemia | 10027496 |
| Atrioventricular block | 10003671 | Miosis | 10027646 |
| Atrioventricular block second degree | 10003677 | Mitochondrial toxicity | 10053961 |
| Azotaemia | 10003885 | Multi-organ failure | 10028154 |
| Back pain | 10003988 | Muscular weakness | 10028372 |
| Bipolar disorder | 10057667 | Musculoskeletal pain | 10028391 |
| Blood cholesterol increased | 10005425 | Musculoskeletal stiffness | 10052904 |
| Blood creatinine increased | 10005483 | Mutism | 10028403 |
| Blood pressure decreased | 10005734 | Myalgia | 10028411 |
| Bone marrow failure | 10065553 | Myocardial infarction | 10028596 |
| Bradycardia | 10006093 | Myocardial ischaemia | 10028600 |
| Bundle branch block left | 10006580 | Myoclonus | 10028622 |
| Cachexia | 10006895 | Myopathy | 10028641 |
| Cardiac arrest | 10007515 | Myositis | 10028653 |
| Cardiac failure | 10007554 | Nail disorder | 10028694 |
| Cardiac failure congestive | 10007559 | Nephrolithiasis | 10029148 |
| Cardiac fibrillation | 10061592 | Nephropathy | 10029151 |
| Cardiomegaly | 10007632 | Nephropathy toxic | 10029155 |
| Cardiotoxicity | 10048610 | Nephrotic syndrome | 10029164 |
| Cerebrovascular accident | 10008190 | Nervousness | 10029216 |
| Chest discomfort | 10008469 | Neuralgia | 10029223 |
| Chills | 10008531 | Neuropathy peripheral | 10029331 |
| Cholelithiasis | 10008629 | Neurotoxicity | 10029350 |
| Cholestasis | 10008635 | Neutropenia | 10029354 |
| Chronic allograft nephropathy | 10063209 | Nightmare | 10029412 |
| Cognitive disorder | 10057668 | Obsessive-compulsive disorder | 10029898 |
| Coma | 10010071 | Oedema | 10030095 |
| Completed suicide | 10010144 | Oliguria | 10030302 |
| Confusional state | 10010305 | Osteopenia | 10049088 |
| Constipation | 10010774 | Overdose | 10033295 |
| Convulsion | 10010904 | Pain | 10033371 |
| Coronary artery disease | 10011078 | Palpitations | 10033557 |
| Cough | 10011224 | Pancreatitis | 10033645 |
| Crying | 10011469 | Pancytopenia | 10033661 |
| Cyanosis | 10011703 | Panic attack | 10033664 |
| Delirium | 10012218 | Panic disorder | 10033666 |
| Delusion | 10012239 | Paraesthesia oral | 10057372 |
| Dementia | 10012267 | Parkinsonism | 10034010 |
| Depression | 10012378 | Peptic ulcer | 10034341 |
| Dermatitis | 10012431 | Peripheral sensory neuropathy | 10034620 |
| Diabetes mellitus | 10012601 | Peripheral vascular disorder | 10034636 |
| Diarrhoea | 10012735 | Pharyngitis | 10034835 |
| Dissociation | 10013457 | Poisoning | 10061355 |
| Dizziness | 10013573 | Polyuria | 10036142 |
| Drug intolerance | 10061822 | Poor quality sleep | 10062519 |
| Drug tolerance | 10052804 | Pregnancy | 10036556 |
| Drug tolerance decreased | 10052805 | Presyncope | 10036653 |
| Dry mouth | 10013781 | Productive cough | 10036790 |
| Duodenal ulcer | 10013836 | Proteinuria | 10037032 |
| Dysarthria | 10013887 | Prothrombin time prolonged | 10037063 |
| Dyslipidaemia | 10058108 | Pruritus | 10037087 |
| Dysphagia | 10013950 | Psoriasis | 10037153 |
| Dyspnoea | 10013968 | Psychosomatic disease | 10049587 |
| Dystonia | 10013983 | Psychotic disorder | 10061920 |
| Electrocardiogram qt interval | 10014385 | Pulmonary toxicity | 10061924 |
| Electrocardiogram qt prolonged | 10014387 | Pyelonephritis | 10037596 |
| Electrocardiogram st segment | 10014389 | Rash | 10037844 |
| Embolism | 10061169 | Renal failure | 10038435 |
| Epistaxis | 10015090 | Renal failure chronic | 10038444 |
| Erectile dysfunction | 10061461 | Renal impairment | 10062237 |
| Erythema | 10015150 | Renal tubular necrosis | 10038540 |
| Erythema multiforme | 10015218 | Restlessness | 10038743 |
| Essential hypertension | 10015488 | Rhabdomyolysis | 10039020 |
| Euphoric mood | 10015535 | Rhinitis | 10039083 |
| Extrapyramidal disorder | 10015832 | Salivary hypersecretion | 10039424 |
| Fatigue | 10016256 | Schizoaffective disorder | 10039621 |
| Fluid retention | 10016807 | Schizophrenia | 10039626 |
| Flushing | 10016825 | Sedation | 10039897 |
| Formication | 10017062 | Serotonin syndrome | 10040108 |
| Gait disturbance | 10017577 | Sexual dysfunction | 10040477 |
| Gastric ulcer | 10017822 | Shock | 10040560 |
| Gastrointestinal haemorrhage | 10017955 | Sinus bradycardia | 10040741 |
| Gastrooesophageal reflux disease | 10017885 | Sinusitis | 10040753 |
| Gingival hyperplasia | 10018283 | Skin toxicity | 10059516 |
| Glomerulonephritis | 10018364 | Sleep disorder | 10040984 |
| Glucose tolerance impaired | 10018429 | Social avoidant behaviour | 10041243 |
| Glycosuria | 10018473 | Somnolence | 10041349 |
| Gout | 10018627 | Stomatitis | 10042128 |
| Graft dysfunction | 10059677 | Stress | 10042209 |
| Graft loss | 10048748 | Sudden cardiac death | 10049418 |
| Graft versus host disease | 10018651 | Sudden death | 10042434 |
| Grand mal convulsion | 10018659 | Suicidal ideation | 10042458 |
| Gynaecomastia | 10018800 | Suicide attempt | 10042464 |
| Haematoma | 10018852 | Syncope | 10042772 |
| Haematuria | 10018867 | Tachycardia | 10043071 |
| Haemolysis | 10018910 | Tardive dyskinesia | 10043118 |
| Haemorrhage | 10055798 | Tension | 10043268 |
| Haemorrhagic diathesis | 10062713 | Thinking abnormal | 10043431 |
| Hallucination | 10019063 | Thrombocytopenia | 10043554 |
| Hemiparesis | 10019465 | Thrombosis | 10043607 |
| Hemiplegia | 10019468 | Thrombotic thrombocytopenic purpura | 10043648 |
| Hepatic cirrhosis | 10019641 | Torsade de pointes | 10044066 |
| Hepatic encephalopathy | 10019660 | Transaminases increased | 10054889 |
| Hepatic enzyme increased | 10060795 | Tremor | 10044565 |
| Hepatic failure | 10019663 | Type 2 diabetes mellitus | 10067585 |
| Hepatic function abnormal | 10019670 | Ulcer | 10045285 |
| Hepatic steatosis | 10019708 | Upper gastrointestinal haemorrhage | 10046274 |
| Hepatitis cholestatic | 10019754 | Urinary incontinence | 10046543 |
| Hepatotoxicity | 10019851 | Urticaria | 10046735 |
| High density lipoprotein decreased | 10020060 | Vasoconstriction | 10047139 |
| Hostility | 10020400 | Ventricular arrhythmia | 10047281 |
| Hot flush | 10060800 | Ventricular extrasystoles | 10047289 |
| Hyperbilirubinaemia | 10020578 | Ventricular failure | 10060953 |
| Hypercalcaemia | 10020583 | Ventricular fibrillation | 10047290 |
| Hyperchlorhydria | 10020601 | Ventricular tachycardia | 10047302 |
| Hypercholesterolaemia | 10020603 | Vision blurred | 10047513 |
| Hyperglycaemia | 10020635 | Visual impairment | 10047571 |
| Hyperhidrosis | 10020642 | Weight decreased | 10047895 |
| Hyperkalaemia | 10020646 | Weight increased | 10047899 |
| Hyperlipidaemia | 10062060 | Withdrawal syndrome | 10048010 |
| Hypersensitivity | 10020751 |  |  |
| Hypertension | 10020772 |  |  |
| Hyperthyroidism | 10020850 |  |  |
| Hypertriglyceridaemia | 10020869 |  |  |
| Hypertrophic cardiomyopathy | 10020871 |  |  |
| Hyperuricaemia | 10020903 |  |  |
| Hypoalbuminaemia | 10020942 |  |  |
| Hypochondriasis | 10020965 |  |  |
| Hypoglycaemia | 10020993 |  |  |
| Hypokalaemia | 10021015 |  |  |
| Hypomagnesaemia | 10021027 |  |  |
| Hypomania | 10021030 |  |  |
| Hyponatraemia | 10021036 |  |  |
| Hypophosphataemia | 10021058 |  |  |
| Hypoprothrombinaemia | 10021085 |  |  |
| Hypotension | 10021097 |  |  |
| Hypothyroidism | 10021114 |  |  |
| Idiopathic thrombocytopenic purpura | 10021245 |  |  |
| Immunodeficiency | 10061598 |  |  |
| Incontinence | 10021639 |  |  |
| Infarction | 10061216 |  |  |

Supplementary 2 PMIDs selected by searching criteria explained in (Table 1 Abstract Inclusion-Exclusion criteria)

| 12952547  16765240  17456828  20112435  20819866  21051745  21156884  22071529  22737097  23449283  23471929  23574801  23740530  24377421  24780720  25024246  25567824  25725597  25827034 | 25991397  26524702  27112538  27969571  28081941  20362271  20504370  20976281  21182790  21722567  21727258  21862746  21931496  23193215  23873756  23913737  23940245  23969148  24177317 | 24297547  24349080  24636641  24658100  24734124  24882379  25104519  25431293  26167484  26209741  26365338  26370823  26725697  26961369  27151343  27621120  27749702  25851993  28222112 |
| --- | --- | --- |

Supplementary 3 Abstracts selected by using the other searching criteria (Not Table 1 Abstract Inclusion-Exclusion criteria)

| **PMID** | **Search Criteria** |
| --- | --- |
| 11388131 | hypertension electronic medical record diagnosis guideline |
| 12461305 | hypertension and "electronic medical record" and algorithm |
| 15323063  22051424  17712071  24283597 | hypertension electronic medical record (code OR retrospective) |
| 15572716  26116311 | myopathy electronic medical record |
| 15758007  20655691 | thrombosis electronic medical record |
| 16567608  17162144  27252874  23439167  23445773 | diabetes electronic medical record |
| 17269833 | electronic medical records adrenal cohort |
| 17567225 | electronic medical records heart failure |
| 22466034 | stroke electronic medical record (cohort* OR retrospective) |
| 27940627 | (cardiotoxicity OR cyanosis OR "peripheral vascular disorder" OR shock OR vasoconstriction OR "hypertrophic cardiomyopathy" OR "acute coronary syndrome" OR "angina pectoris" OR "cardiac arrest" OR infarction OR ischaemia OR" myocardial infarction" OR "myocardial ischaemia" OR "sudden cardiac death" OR arteriosclerosis OR "coronary artery disease" OR arrhythmia OR "atrial fibrillation" OR "atrioventricular block" OR "atrioventricular block second degree" OR bradycardia OR "bundle branch block left" OR "cardiac fibrillation" OR "electrocardiogram st segment" OR palpitation* OR presyncope OR "sinus bradycardia" OR syncope OR tachycardia OR "ventricular arrhythmia" OR "ventricular extrasystoles" OR "ventricular failure" OR "ventricular fibrillation" OR "ventricular tachycardia" OR "torsade de pointes" OR electrocardiogram qt interval OR" electrocardiogram qt prolonged" OR "cardiac failure" OR cardiac failure congestive OR cardiomegaly OR "blood pressure decreased" OR "blood pressure increased" OR thrombotic thrombocytopenic purpura OR "sudden death") electronic health record (cohort OR surveillance or case-control or epidemiological or Longitudinal Studies) (code or billing or algorithm)(code OR codes OR algorithm* or case definition) |
| 23781409 | (Diabetes type II) AND ("Electronic Health record" OR "Electronic Medical Record" OR "Electronic Health records" OR "Electronic Medical Records") AND validation |
| 23968235 | electronic health records and anemia |
| 24303267 | (diabetes mellitus OR glucose tolerance impaired OR glycosuria OR hyperglycaemia OR hypoglycaemia OR type 2 diabetes mellitus OR DM2) electronic health record (cohort OR surveillance or case-control or epidemiological or Longitudinal Studies) (code or billing or algorithm) (code OR codes OR algorithm* or case definition) |
| 24507049 | renal failure electronic medical record |
| 25091637 | myocardial infarction electronic medical record (retrospective OR cohort*) |
| 25933736 | electronic medical record Arrhythmia cohort |
| 26221186 | electronic medical record Arrhythmia algorithm |
| 26283069 | electronic health records and anemia and validation |
| 27317850 | (drug-induced OR "adverse events" OR "DDI" OR drug drug interaction OR "adverse reaction") electronic health record (cohort OR surveillance or case-control or epidemiological or Longitudinal Studies) |
| 26082655 | (asthenia OR chest discomfort OR chills OR dry mouth OR dysphagia OR fluid retention OR flushing OR formication OR haematoma OR hot flush OR hypercalcaemia OR hyperkalaemia OR hypokalaemia OR hypomagnesaemia OR hyponatraemia OR hypophosphataemia OR mitochondrial toxicity OR multi-organ failure OR oedema OR overdose OR pain OR poisoning) electronic health record (cohort OR surveillance or case-control or epidemiological or Longitudinal Studies) (code or billing or algorithm)(code OR codes OR algorithm* or case definition) |

Supplementary 4 Annotation guidelines to annotate a phenotype definition in the literature

| **Inclusion category (five dimensions)** |
| --- |
| **Inclusion dimension 1 – Biomedical & Procedure:**  Criteria 1: The sentence should include two entities:  Rule for criteria 1: [Biomedical\|Procedrue] AND [Definition criteria]   1. [Biomedical\|Procedrue]: any biomedical or procedure terms, or any of the following: disease stages, symptoms, outcome of interest, diseases, laboratory & vital tests, diagnosis, procedure, clinical observation, person-time, Bed rest, Height, race, comorbidity, weight, sex or gender (Males, females, women, woman, man, men), hospitalization, birth date, surgery, chronic condition, BMI, age, ADE(drug-induced side effect), medication adherence, drug intolerance, cell level (gene/allele/SNPs/ homozygotes/dna). 2. [Definition criteria]: can be any of the following:  - Verbs to define a phenotype: defined, identify, identified, included, excluded, calculate, having, undergoing, underwent, who had, documented, diagnosed, classified, consider, selected, counted, captured - Nouns to define a phenotype: inclusion, exclusion, definition, case identification, eligible, presence, criteria, Algorithm, diagnostic criteria, presence, absence, parameter, incident, sign, history, diagnosis, diagnoses, initiation, onset, occurrence, referral, guideline, category or categories, stage, outcome, outcome of interest, history, endpoint, examination, severity, adverse event, condition of interest - Phrase: “Patients/case/subject/child with”, “Patients/case/subject/child had”, “Patients/case/subject/child who”, “primary diagnosis”, “secondary diagnosis”, “primary procedure”, “secondary procedure”, “based on”, evidence of.   Criteria 2: Definitions in table, figure, box, or appendix. The sentence provide evidence of a phenotype definition information presented in other sources, rather than text, such as table, figure, box, or appendix:  Rule for criteria 2: [Table terms] AND [Definition terms]   1. [Table terms]: Table, figure, Box, appendix. 2. [Definition terms]: inclusion, exclusion, definition, case identification, inclusion criteria, criteria, phenotyping algorithm, exclusion criteria.   Examples for inclusion dimension 1:   - “[identification] of [syndromic conditions]” (PMID17567225) – criteria 1 - “Such phenocopies [include] several [vasculitides, Buerger’s disease, embolism, trauma to leg arteries and other rare arteriopathies]”. (PMID20819866) – criteria 1 - “We first [calculated] the prevalence of [prehypertension], [stage] 1 [hypertension], and stage 2 [hypertension] in the cohort”. (PMID17712071) – criteria 1 with categories or stages - “The [categories] of [race] were ‘white’, ‘black or African American’, ‘American Indian or Alaskan’, ‘Asian’, ‘other’, and ‘unknown’” (PMID20819866) – criteria 1 with categories or stages - “ Six comorbid disease conditions were selected and validated using the definitions reported in Table 3”. (PMID21051745) – criteria 2 - “The diagnoses are presented in hierarchical order in the first column of the Table”. (PMID23449283) – criteria 2   **Inclusion dimension 2 – Standard codes:**  Criteria 1: Mention of standard terms (e.g. International classification of diseases), such as ICD, CPT, UMLS, SNOMED, RxNorm, billing code, Read codes, diagnostic code¸ procedure code. Accepted formats: Short or long form (ICD or International Classification of Diseases) or list diagnostic or procedure codes.  Criteria 2: able terms with evidence of diagnostic or procedure codes list/ code definitions/algorithms: Table, figure, Box, appendix  Example for inclusion dimension 2:   - “a primary or any secondary discharge diagnosis (International Classification of Diseases, Ninth Revision, Clinical Modification [ICD-9-CM] code) of myoglobinuria (791.3)” (PMID15572716)   **Inclusion dimension 3 – Medications:**  Criteria 1: Keywords describe medications: e.g. generic drug names, prescribing, medication regimens, recommended agent, medication prescribed, drug dosage, drug frequency, drug route, medications, prescriptions.  Criteria 2: Drug name o-occurs with any of the following: medication, dose, treatment, therapy, drug, receiving, received, prescrib, using, use, use of, inclusion, include, exclusion, exclude, definition, case identification, identify, eligible, presence, criteria, presence, initiation, window, dose, guideline, history, started, agent(s), drug, medication, exposure, who had, treated with, indication, drug dosage, drug frequency, drug route, cohort.  Criteria 3: Medication terms co-occur with table, figure, box, or appendix: Table terms with evidence of a list of medication terms (Table, figure, Box, appendix).  Examples for inclusion dimension 3:   - “Other risk factors and comorbidities were ascertained based on ICD-9-CM codes, medication use and laboratory data”. (PMID20819866) – criteria 1 - “Prior antihypertensive therapy was deﬁned as the use of any AHDs before the start of amlodipine, which were not discontinued on or before the start of amlodipine therapy”. (PMID15323063) – criteria 2 - “Table 1 outlines the recommended agents for specific comorbid conditions, as stated in our guideline”. (PMID12952547)- criteria 3   **Inclusion dimension 4 – Laboratories:**  Criteria 1: The sentence should provide evidence of using clinical measurable values (i.e. laboratory values, vital values, procedures, clinical) combined with real values. The sentence should include [Clinical or procedure] AND [Measurable value]:   1. [Clinical or procedure]: Clinical can be any of the following: disease stages, symptoms, outcome of interest, diseases, laboratory & vital tests, diagnosis, procedure, clinical observation, Height, weight, BMI, age. 2. [Measurable value]: Any of the following:  - Terms or numbers indicate measurable values: >, <, ≥, numerical values, more than, less than. - Other = ["mg", "ml”, “mg/dL”, “years old”] - Other clue words combined with real values: value, measure, measurement, reference range, normal range, reading, level.   Example for inclusion dimension 4:   - “Achievement of lipid goals was deﬁned as recommended by the National Cholesterol Education Program Adult Treatment Panel III guidelines16 as follows: LDL-C less than 100 mg/dL, triglyceride level less than 150 mg/dL, HDL-C greater than 40 mg/dL, and non–HDL-C less than 130 mg/dL”. (PMID16765240)   **Inclusion dimension 5 – Use of Natural Language Processing (NLP):**  Criteria 1: The sentence provides evidence of using NLP in a phenotype definition.  Rule for criteria 1: [Phenotype, procedure, medication] AND [NLP terms]   1. [Phenotype, procedure, medication]: Terms can be any of the following: disease stages, symptoms, outcome of interest, diseases, diagnosis, procedure, clinical observation, drugs, medications. Other terms can be considered: person-time, Bed rest, Height, race, comorbidity, weight, sex, birth date, surgery, diseases, signs/symptoms, anatomical sites, procedure, drug, medication. 2. [NLP terms]: Natural Language processing, Natural language, nlp, text mining, "wildcard character", “bag of words”, parses, “named entity”, rule-based, NLP algorithm, n-grams, Regular Expression, tokenization, normalization, stemming, Lemmatization, named entity, named entity recognition (NER).   Criteria 2: NLP evidence in a phenotype definition and this information explained in a table, figure, box, or appendix.  Rule for criteria 2: [NLP terms] AND [Table terms]   1. [NLP terms]: Natural Language processing, Natural language, NLP, text mining, "wildcard character", “bag of words”, parses, “named entity”, rule-based, NLP algorithm, n-grams, Regular Expression, tokenization, normalization, stemming, Lemmatization, Named entity, synonym, Named entity recognition (NER), nonnegated. 2. [Table terms]: Table, figure, Box, appendix.   Examples for inclusion dimension 5:   - “Example of a Clinical Note Represented as a “Bag of Words” . . . HF status positive negative Covariate #1 "heart" 3 1 Covariate #2 "pulmonary"” (PMID17567225) – criteria 1 - “Rule-based and machine learning techniques were applied to clinical narratives and smoking status was classiﬁed as ‘past’, ‘current’, ‘smoker’, ‘non-smoker’, or ‘unknown’”. (PMID20819866) – criteria 1 - “Structuring free text into useable coded data Text mining techniques were used to code diagnoses and drug prescriptions into ICD10 and ATC classiﬁcation systems, respectively”. (PMID26209741) – criteria 1 medications - “Details of text mining for identifying diagnoses are contained in a supplementary technical document” (PMID26209741) – criteria 2 |
| **Intermediate category (two dimensions)** |
| **Intermediate dimension 1 – Data sources:**  Criteria 1: mention of used sources.   - Electronic health records keywords: Electronic health records, EHR, electronic medical record, EMR, database, registry, biobank, biospecimen, biorepositories   Criteria 2: mention of datatypes/variables used in EHR/EMR.   - Medical records keywords: progress notes, clinical notes/reports, laboratory records/data, radiology report/data, pharmacy records/data, administrative records/data, insurance claims/records/data, patient record, patient chart, hard copy report, medical chart, computerized charts. - Clinical data keywords: referral, encounter, immunization, consultation report, laboratory, dismissal summaries, Height measurement, weight measurement, genetic data, serological data, problem list, scanned image, free-text, diagnoses list - Procedure data keywords: claim, discharge, hospitalization, visit, admission, outpatient, inpatient, billing, hospital report, note. - Other data mentions with evidence in EHR/EMR: demographic, sociodemographic, patient characteristics, abnormal measurement, follow-up data/measurement., encounter identifier.   Criteria 3: Any clinical or procedure followed with data keywords.  Rule for criteria 3: [Biomedical\|Procedrue] AND [Data keywords]   1. [Biomedical\|Procedrue]: any biomedical or procedure terms, or any of the following: disease stages, symptoms, outcome of interest, diseases, laboratory & vital tests, diagnosis, procedure, clinical observation, person-time, Bed rest, Height, race, comorbidity, weight, sex or gender (Males, females, women, woman, man, men), hospitalization, birth date, surgery, chronic condition, BMI, age, ADE(drug-induced side effect), medication adherence, drug intolerance, cell level (gene/allele/SNPs/ homozygotes/dna). 2. [Data keywords]: data, measures, measurement, value, values, datamart, dataset.   Examples for intermediate dimension 1:   - “Computerized medical and pharmacy records were reviewed for patient demographics, antihypertensive medications, comorbid conditions, and BP readings”. (PMID11388131) – criteria 1 & 2 - “The data were all based on pharmacy claim records from the KP electronic prescription system”. (PMID17269833) – criteria 1 & 2 - “This detailed information includes medical history, clinical assessments, consultation reports, dismissal summaries, laboratory and radiology results, and correspondence”. (PMID17162144) – criteria 2   **Intermediate dimension 2 – Study design or Institutional Review Board (IRB):**  Criteria 1: Institutional Review Board (IRB) or Study design. If any of the following is in the sentence:   - Study design keywords: Retrospective, observational study, longitudinal study, case-control study, “random/ly sample/d”, Inception cohorts, matched controls, matched cases, intervention group, control group, matched pairs, case-control pairs, cohort, negative cohort, positive cohort, pilot study, stratified, stratification, prospective, Surveillance Study, control. - Gold standard keywords: chart review, manual review, notes reviewed, records reviewed, manual abstraction, expert panel, validation study, gold standard, standardized abstraction, standardized protocol. - IRB keywords: IRB, Institutional Review Board, study protocol   Criteria 2: Any of the following co-occurrences the same sentence:   - “case” and “control” - “chart” and “review”, “record” and “review”, or similar.   Examples for intermediate dimension 2s:   - “IRB approval The Institutional Review Board (IRB) at the Birmingham VA Medical Center approved this study”. (PMID24377421) – criteria 1 - “STUDY DESIGN: Retrospective chart review”. (PMID11388131) – criteria 1 - “To establish the control group, all active patients in the practice for less than 12 months were excluded”. (PMID11388131) – criteria 1 - “Chart review Confirmation of case status by manual review” (PMID12952547) – criteria 1 |
| **Exclusion category (three dimensions)** |
| **Exclusion dimension 1 – Irrelative evidence:**  Criteria 1: Evidence of information relevant to other components of the study that might not assist in phenotyping. Each of the following sub-dimensions shows examples of keywords:   - Physical location (geographic) of the study only: information about the location (country, county, city, zip code, region, geographic).   Note: We exclude from this criterion general location names because it can cause ambiguity with other institution names that are not physical location. Examples: institute, office, clinic, department…etc. In addition, we exclude from this criterion: if the state name is referring to the hospital.   - Ethical: consent, ethics, patient approval, patient denial, HIPAA. - Financial: Funding, financial support, copayment, charged, sponsor, cost, insurance coverage, fee-per-service. - Patient direct contact or enrollment: The sentence that shows evidence of a direct contact or enrollment of patients in the study. Example keywords: Surveys, questionnaire, interviews, instructions, recruitment, recruit, enrollment, enroll patients, 9-item Patient Health Questionnaire (PHQ-9) - Providers & researchers: provider, physician, medical student, nurse, team, staff, clinician, fellow, --ologist, resident, general practitioner (GP), team, psychiatric, principal investigator, case manager.   Note: We exclude from this criterion: author   - Performance: performance evaluation, training, performance measure, CPOE intervention, human error - Quality of care: Quality of care, Quality Assurance, Quality Improvement   Examples for exclusion dimension 1:   - “Patients from the Department of Neurology, the Newborn Service, and the Neonatal Intensive Care Unit were excluded, as were patients receiving mechanical or pharmacologic prophylaxis”. (PMID15758007) – (Type: Location) - “Reasons for exclusion were as follows: 6 persons denied permission to use their medical records for research” (PMID17162144) – (Type: Ethical) - “All patients were members of the managed care system and incurred a signiﬁcant ﬁnancial advantage from having their prescriptions ﬁlled within the system”. (PMID16765240) – (Type: Financial) - “Patients overdue for specific screening services received personalized letters recommending the needed service (e.g., cholesterol testing or dilated eye examinations) on a quarterly basis”. (PMID16567608) – (Type: Patient direct contact or enrollment) - “Physicians received training on the use of the electronic medical record system and associated tools, such as reminders, from consultants working for the vendor company”. (PMID16567608) – (Type: Provider) - “Physicians received training on the use of the electronic medical record system and associated tools, such as reminders, from consultants working for the vendor company”. (PMID16567608) – (Type: Performance) - “Quality of care was determined by measuring the same parameters designed to measure the awareness, treatment, and control of hypertension”. (PMID12461305) – (Type: Quality of care)   **Exclusion dimension 2 – Computational and statistical evidence:**  Criteria 1: Evidence of information relevant to computational and statistical that might not assist in phenotyping. Each of the following sub-dimensions shows examples of keywords:   - Alerts: computer alerts, reminders, intranet tracking, continuously updated, robust, automated - Software or tool: software, platform, plugin, computer, tool - Statistical methods (usually toward the end of the method section). Any of the following statistical terms (or similar):  \| - Analysis of covariance (ANCOVA) \| - Measure (measured) - verb \| \| --- \| --- \| \| - Area under the receiver operating characteristic curves (AUC) \| - Model (modeled, modeling) \| \| - Bayes \| - Multiplication [ x ] \| \| - Bias \| - Multivariate \| \| - Bivariate \| - Normally distributed \| \| - Calculate (calculated, calculates, calculations) \| - Odds \| \| - Charlson’s comorbidity index \| - Over-fitting \| \| - Chisquare \| - P value \| \| - Chi-square \| - Package \| \| - Cluster \| - Permutation \| \| - Coefficient \| - Poisson distribution \| \| - Compute (computed, computes) - verb \| - Poisson regression \| \| - Confidence interval, CI \| - Predict \| \| - Correlation \| - Predicted \| \| - Covariance \| - Predictive value \| \| - Cox \| - Probability \| \| - Degrees of freedom \| - Propensity score \| \| - Descriptive statistics \| - R statistical language \| \| - Equation \|  \| \| - Fisher exact test \| - Regression \| \| - Fisher’s exact \| - Relative risk (rr) \| \| - Fishers test \| - Risk score \| \| - General linear model \| - SAS \| \| - Goodness-of-fit \| - Sensitivity, specificity \| \| - Graphic \| - Simulation \| \| - Imputat \| - SPSS \| \| - Independent samples t-test \| - Statistically significant \| \| - Kaplan-meier \| - Statistics, statistical \| \| - Kolmogorov– smirnov \| - T-test \| \| - Likelihood \| - Two-tailed \| \| - Logistic \| - Univariate statistical analysis \| \| - Logistic regression \| - Variance \| \| - Mantel–Cox (log-rank) \| - Welch and Brown–Forsythe \| \| - Mean, median, mode \| - Weighted \| \| - Structured Query Language (SQL) \|  \|   Examples for exclusion dimension 2:   - “We used logistic regression models with generalized estimating equations to adjust for race, year, race x year interactions, age, and sex”. (PMID16567608) – (Type: Statistics) - “We used the proportional-hazards model to estimate the relative hazard of clinical end points associated with the computer alert and obtained 95 percent confidence intervals from this model”. (PMID15758007) – (Type: Alerts)   **Exclusion dimension 3 – Insufficient evidence:**  Criteria 1: Sentences with insufficient evidence. We mean by insufficient evidence is a sentence that does not met any of the dimensions in all categories (inclusion, intermediate, exclusion 1 & 2).  Example of exclusion dimension 3:   - “BSA= beclomethasone-salmeterol; COPD= chronic obstructive pulmonary disease; FSA= fluticasone-salmeterol; ICS= inhaled corticosteroid”. (PMID17162144) |

Supplementary 5 Entities and terms in the 86 abstracts using PubTator annotation tool

| **Entity** | **Term** | **Count of PMID** | |  |
| --- | --- | --- | --- | --- |
| **Chemical** | | | **60** | |
|  | 1RA | 1 | |  |
|  | alcohol | 1 | |  |
|  | aminosalicylates | 1 | |  |
|  | amlodipine | 1 | |  |
|  | amlodipine besylate | 1 | |  |
|  | atorvastatin | 1 | |  |
|  | beclomethasone | 1 | |  |
|  | bilirubin | 1 | |  |
|  | calcium | 1 | |  |
|  | cerivastatin | 1 | |  |
|  | cerivastatin-fibrate | 1 | |  |
|  | chloride | 1 | |  |
|  | cholesterol | 6 | |  |
|  | creatinine | 3 | |  |
|  | Cys | 1 | |  |
|  | cystatin C | 1 | |  |
|  | DVT | 1 | |  |
|  | fatty acid | 1 | |  |
|  | ferritin | 1 | |  |
|  | fibrate | 1 | |  |
|  | fluticasone | 1 | |  |
|  | gabapentin | 1 | |  |
|  | glucose | 1 | |  |
|  | Hg | 1 | |  |
|  | irbesartan | 1 | |  |
|  | iron | 2 | |  |
|  | lisinopril | 1 | |  |
|  | losartan | 1 | |  |
|  | N | 1 | |  |
|  | Neurontin | 1 | |  |
|  | olmesartan | 1 | |  |
|  | PIO | 1 | |  |
|  | potassium | 1 | |  |
|  | PPV | 2 | |  |
|  | pravastatin | 1 | |  |
|  | rivaroxaban | 1 | |  |
|  | SABA | 1 | |  |
|  | salmeterol | 1 | |  |
|  | serotonin | 1 | |  |
|  | simvastatin | 1 | |  |
|  | statin-fibrate | 1 | |  |
|  | statins | 1 | |  |
|  | steroid | 1 | |  |
|  | TGL | 1 | |  |
|  | thiopurines | 1 | |  |
|  | triamcinolone acetonide | 1 | |  |
|  | triglyceride | 1 | |  |
|  | triheptanoin | 1 | |  |
|  | uric acid | 1 | |  |
|  | valsartan | 1 | |  |
|  | venlafaxine | 1 | |  |
| **Disease** | | | **264** | |
|  | AAA | 1 | |  |
|  | abdominal aortic aneurysm | 1 | |  |
|  | acute gout, chronic gout | 1 | |  |
|  | acute kidney injury | 1 | |  |
|  | acute liver failure | 1 | |  |
|  | acute myocardial infarction | 1 | |  |
|  | acute renal failure | 2 | |  |
|  | adult-onset asthma | 1 | |  |
|  | agranulocytosis | 1 | |  |
|  | AHDs | 1 | |  |
|  | ALD | 1 | |  |
|  | allergic reaction | 1 | |  |
|  | allergic reactions | 1 | |  |
|  | allergies | 2 | |  |
|  | allergy | 1 | |  |
|  | AMI | 1 | |  |
|  | anemia | 1 | |  |
|  | aneurysm | 1 | |  |
|  | anxiety | 1 | |  |
|  | anxiety symptoms | 1 | |  |
|  | AOA | 1 | |  |
|  | AOA to infection | 1 | |  |
|  | ARDS | 1 | |  |
|  | ARF | 1 | |  |
|  | ARI | 1 | |  |
|  | arrhythmia | 1 | |  |
|  | arthritis | 1 | |  |
|  | ASCVD | 1 | |  |
|  | aspiration | 1 | |  |
|  | asthma | 3 | |  |
|  | atherosclerotic | 1 | |  |
|  | Atrial Fibrillation | 1 | |  |
|  | beta-lactams | 1 | |  |
|  | bipolar disorder | 1 | |  |
|  | bleeding | 3 | |  |
|  | BP reduction | 1 | |  |
|  | breast cancer | 1 | |  |
|  | CAD | 1 | |  |
|  | cancer | 3 | |  |
|  | cancers | 1 | |  |
|  | cardiomyopathy | 1 | |  |
|  | cardiovascular disease | 1 | |  |
|  | Cardiovascular Health Study | 1 | |  |
|  | CAS | 1 | |  |
|  | catheter-directed thrombolysis | 1 | |  |
|  | CDT | 1 | |  |
|  | cellulitis | 1 | |  |
|  | cerebrovascular disease | 1 | |  |
|  | CHD | 2 | |  |
|  | CHF | 1 | |  |
|  | chronic disease | 1 | |  |
|  | chronic diseases | 1 | |  |
|  | chronic kidney disease | 1 | |  |
|  | chronic obstructive pulmonary disease | 1 | |  |
|  | CKD | 3 | |  |
|  | CLIA | 1 | |  |
|  | cognitive impairment | 1 | |  |
|  | congestive heart failure | 1 | |  |
|  | COPD | 1 | |  |
|  | coronary heart disease | 3 | |  |
|  | CRC | 1 | |  |
|  | Crohn disease | 1 | |  |
|  | CRT-D | 1 | |  |
|  | CVD | 1 | |  |
|  | death | 2 | |  |
|  | deep vein thrombosis | 1 | |  |
|  | deep-vein thrombosis | 1 | |  |
|  | defined as high blood pressure | 1 | |  |
|  | dementia | 2 | |  |
|  | depression | 1 | |  |
|  | Device failures | 1 | |  |
|  | diabetes | 16 | |  |
|  | diabetes care | 1 | |  |
|  | diabetes mellitus | 8 | |  |
|  | diabetic | 3 | |  |
|  | diabetics | 1 | |  |
|  | DM | 4 | |  |
|  | DVT | 1 | |  |
|  | ectopic pregnancies | 1 | |  |
|  | epilepsy | 1 | |  |
|  | GAD | 1 | |  |
|  | generalized anxiety disorder | 1 | |  |
|  | GI and other bleeding complications | 1 | |  |
|  | GI bleeds | 1 | |  |
|  | gout | 2 | |  |
|  | gout flares | 1 | |  |
|  | gout-related visits | 1 | |  |
|  | heart disease | 1 | |  |
|  | heart failure | 7 | |  |
|  | Hemorrhage | 1 | |  |
|  | HEP | 1 | |  |
|  | Hepatic encephalopathy | 1 | |  |
|  | hepatocellular carcinoma | 1 | |  |
|  | HF | 2 | |  |
|  | HH | 1 | |  |
|  | hip fractures | 1 | |  |
|  | HLD | 1 | |  |
|  | HLMs | 1 | |  |
|  | HSD | 1 | |  |
|  | HTN | 1 | |  |
|  | hyperkalemia | 1 | |  |
|  | hypertension | 11 | |  |
|  | hypertensive | 3 | |  |
|  | hypertensives | 1 | |  |
|  | hypoglycemia | 1 | |  |
|  | hyporesponsive | 1 | |  |
|  | hyporesponsiveness | 1 | |  |
|  | IBD | 1 | |  |
|  | ICD | 2 | |  |
|  | ICS | 1 | |  |
|  | IDA | 1 | |  |
|  | IHD | 1 | |  |
|  | iliofemoral DVT | 1 | |  |
|  | incremental systolic BP reduction | 1 | |  |
|  | infection | 1 | |  |
|  | injury research | 1 | |  |
|  | injury type definitions | 1 | |  |
|  | injury types | 1 | |  |
|  | ischaemic heart disease | 1 | |  |
|  | LBBB | 1 | |  |
|  | LE PAD | 1 | |  |
|  | major bleeding | 1 | |  |
|  | MB | 1 | |  |
|  | MDD | 2 | |  |
|  | MELD | 1 | |  |
|  | mineral abnormalities | 1 | |  |
|  | Model for End-Stage Liver Disease | 1 | |  |
|  | myocardial infarction | 2 | |  |
|  | nephrolithiasis | 1 | |  |
|  | neutropenia | 1 | |  |
|  | neutrophilia | 1 | |  |
|  | NVAF | 1 | |  |
|  | obese | 1 | |  |
|  | obesity | 3 | |  |
|  | osteoarthritis | 3 | |  |
|  | PAD | 2 | |  |
|  | pain | 1 | |  |
|  | pancreatitis | 1 | |  |
|  | parkinsonism | 1 | |  |
|  | peripheral arterial disease | 1 | |  |
|  | pneumonia | 3 | |  |
|  | poisoning | 1 | |  |
|  | postoperative complications | 1 | |  |
|  | postoperative myocardial infarction | 1 | |  |
|  | prehypertension | 1 | |  |
|  | Preoperative anemia | 1 | |  |
|  | pulmonary embolism | 2 | |  |
|  | RBBB | 1 | |  |
|  | reduced kidney function | 1 | |  |
|  | rhabdomyolysis | 2 | |  |
|  | rheumatoid arthritis | 2 | |  |
|  | right bundle branch block | 2 | |  |
|  | rupture | 1 | |  |
|  | SCD | 1 | |  |
|  | Scotia | 1 | |  |
|  | sepsis | 2 | |  |
|  | SIRS | 1 | |  |
|  | SSS | 1 | |  |
|  | stroke | 2 | |  |
|  | systemic inflammatory response syndrome | 1 | |  |
|  | T2D | 2 | |  |
|  | TBI | 1 | |  |
|  | TBI-related condition | 1 | |  |
|  | thrombosis | 1 | |  |
|  | thrombus | 1 | |  |
|  | tophaceous gout | 1 | |  |
|  | trauma | 1 | |  |
|  | TSAT | 2 | |  |
|  | tumor | 1 | |  |
|  | type | 3 | |  |
|  | type 2 diabetes | 3 | |  |
|  | type of injury | 1 | |  |
|  | UC | 1 | |  |
|  | ulcer prophylaxis | 1 | |  |
|  | Ulcerative Colitis | 1 | |  |
|  | ULT | 1 | |  |
|  | urate-lowering therapy | 1 | |  |
|  | venous thromboembolism | 1 | |  |
|  | weight loss | 1 | |  |
|  | white-black disparity | 1 | |  |
| **Gene** | | | **18** | |
|  | ACE | 1 | |  |
|  | Angiotensin-converting enzyme | 1 | |  |
|  | ARNO | 1 | |  |
|  | CLNK | 1 | |  |
|  | eGFR | 1 | |  |
|  | Epoetin | 1 | |  |
|  | HFE | 1 | |  |
|  | HSD | 1 | |  |
|  | K77 | 1 | |  |
|  | KCNH2 | 1 | |  |
|  | LDLR | 1 | |  |
|  | RYR2 | 1 | |  |
|  | serotonin transporter | 1 | |  |
|  | transferrin | 3 | |  |
|  | zip | 1 | |  |
|  | 3/5/2019 | 1 | |  |
| **Mutation** | | | **3** | |
|  | Cys282Tyr | 1 | |  |
|  | His63Asp | 1 | |  |
|  | p.Cys282Tyr | 1 | |  |
